# Integrated analyses of metabolomics and transcriptomics reveal the potential regulatory roles of long non-coding RNAs in gingerol biosynthesis

**DOI:** 10.1186/s12864-023-09553-5

**Published:** 2023-08-26

**Authors:** Wenlin Zhang, Yang Yang, Xuedong Zhu, Suyu Yang, Ximei Liao, Honglei Li, Zhexin Li, Qinhong Liao, Jianmin Tang, Guohua Zhao, Lin Wu

**Affiliations:** 1 Chongqing Key Laboratory of Economic Plant Biotechnology, College of Landscape Architecture and Life Science/Institute of Special Plants, Chongqing University of Arts and Sciences, Yongchuan, 402160 China; 2 College of Food Science, Southwest University, Beibei, 400715 China; 3 Chongqing Key Laboratory of Biology and Genetic Breeding for Tuber and Root Crops, College of Agronomy and Biotechnology, Southwest University, Beibei, 400715 China; 4 Southeast Chongqing Academy of Agricultural Sciences, Fuling, 408000 China

**Keywords:** Ginger, Metabolomics, Transcriptomics, Long non-coding RNAs, Gingerol biosynthesis

## Abstract

**Background:**

As the characteristic functional component in ginger, gingerols possess several health-promoting properties. Long non-coding RNAs (lncRNAs) act as crucial regulators of diverse biological processes. However, lncRNAs in ginger are not yet identified so far, and their potential roles in gingerol biosynthesis are still unknown. In this study, metabolomic and transcriptomic analyses were performed in three main ginger cultivars (leshanhuangjiang, tonglingbaijiang, and yujiang 1 hao) in China to understand the potential roles of the specific lncRNAs in gingerol accumulation.

**Results:**

A total of 744 metabolites were monitored by metabolomics analysis, which were divided into eleven categories. Among them, the largest group phenolic acid category contained 143 metabolites, including 21 gingerol derivatives. Of which, three gingerol analogs, [8]-shogaol, [10]-gingerol, and [12]-shogaol, accumulated significantly. Moreover, 16,346 lncRNAs, including 2,513, 1,225, and 2,884 differentially expressed (DE) lncRNA genes (DELs), were identified in all three comparisons by transcriptomic analysis. Gene ontology enrichment (GO) analysis showed that the DELs mainly enriched in the secondary metabolite biosynthetic process, response to plant hormones, and phenol-containing compound metabolic process. Correlation analysis revealed that the expression levels of 11 DE gingerol biosynthesis enzyme genes (GBEGs) and 190 transcription factor genes (TF genes), such as *MYB1*, *ERF100*, *WRKY40*, etc. were strongly correlation coefficient with the contents of the three gingerol analogs. Furthermore, 7 and 111 upstream *cis*-acting lncRNAs, 1,200 and 2,225 upstream *trans*-acting lncRNAs corresponding to the GBEGs and TF genes were identified, respectively. Interestingly, 1,184 DELs might function as common upstream regulators to these GBEGs and TFs genes, such as *LNC_008452*, *LNC_006109*, *LNC_004340,* etc. Furthermore, protein–protein interaction networks (PPI) analysis indicated that three TF proteins, MYB4, MYB43, and WRKY70 might interact with four GBEG proteins (PAL1, PAL2, PAL3, and 4CL-4).

**Conclusion:**

Based on these findings, we for the first time worldwide proposed a putative regulatory cascade of lncRNAs, TFs genes, and GBEGs involved in controlling of gingerol biosynthesis. These results not only provide novel insights into the lncRNAs involved in gingerol metabolism, but also lay a foundation for future in-depth studies of the related molecular mechanism.

**Supplementary Information:**

The online version contains supplementary material available at 10.1186/s12864-023-09553-5.

## Background

Ginger (*Zingiber officinale* Roscoe), a perennial herb belonging to the *Zingiberaceae* family, has been widely used worldwide as a spice and medicine for over 2,000 years [1, 2]. Ginger contains more than 60 bioactive compounds, such as phenolic compounds, terpenes, polysaccharides, lipids, and raw fibers. Among them, phenolic compounds, especially gingerols, representing a series of phenol analogs consisting of shogaols, paradols, and gingerone, are the characteristic health-promoting constituent for ginger [2, 3]. Notably [6]-gingerol is of higher abundance component [2, 4]. For example, Jiang et al. monitored the contents of gingerols in ginger rhizomes by liquid chromatography-tandem mass spectrometry (LC–MS) analysis. The results exhibited that [6]-gingerol was the major constituent of gingerols with a mean value of 195.87 μg/g; whereas the contents of other gingerols, such as [8]-gingerol and [10]-gingerol, were much lower [5]. Now, clinical studies have shown that gingerols, specifically [6]-gingerol, are promising in the treatment of degenerative disorders, vomiting, and cancer [6]. Additionally, gingerols are also proved to exhibit anti-inflammatory and antioxidant properties [7]. Therefore, gingerols possess important medicinal value.

Recently, based on available findings, we proposed a backbone biosynthetic pathway for gingerols based on chromosome-scale reference genome assembly, metabolomics, and transcriptome analysis [2]. Briefly, this complicated pathway is composed of two consecutive steps and 12 gingerol biosynthesis enzyme gene (GBEG) families involved in phenylalanine ammonia lyase gene (*PAL*), cinnamate 4-hydroxylase gene (*C4H*), 4-coumarate-CoA ligase gene (*4CL*), p-coumaroyl shikimate transferase gene (*CST*), p-coumaroyl 5-O-quinate/shikimate 3’-hydroxylase gene (*C3’H*), caffeic acid 3-O-methyltransferase gene (*C3OMT*), caffeoyl-CoA O-methyltransferase gene (*CCOMT*), caffeoylshikimate esterase gene (*CSE*), polyketide synthase gene (*PKS*), NADPH-dependent alkanal/one oxidoreductase gene (*AOR*), dehydrogenase gene (*DHN*), and dehydratase gene (*DHT*) [2]. The first part is mainly composed of the catalysis the formation of feruloyl-CoA catalyzed from L-phenylalanine, which is also the main synthesis pathway of lignin or anthocyanins [8, 9]. Therefore, the enzymes that participate in this process are relatively conserved in most plants [2]. The specific enzymes to the *Zingiberaceae* family, such as PKS, AOR, DHN, and DHT, are involved in the second steps of the pathway and contribute to the catalysis the formation of gingerols from feruloyl-CoA [2, 10]. Meanwhile, transcription factors (TFs) from several families are involved in controlling gingerols biosynthesis, such as v-myb avian myeloblastosis viral oncogene homolog (MYB), basic leucine zipper (bZIP), DNA binding with one finger (DOF), and basic helix-loop-helix (bHLH) [2, 4].

Long non-coding RNA genes (lncRNAs), previously considered as expression noise of protein-coding genes, are a class of transcripts with a length > 200 bp and lack of coding ability [11, 12]. Notably, lncRNAs are primarily divided into three categories according to their location in the genome, including long intergenic lncRNAs (lincRNAs), intronic lncRNAs, and antisense lncRNAs [13]. With the development of next-generation sequencing technologies, abundant lncRNAs have been identified in diverse plant species, such as melon (*Cucumis melo* L.), kiwifruit (*Actinidia chinensis*), rice (*Oryza sativa*), Chinese plum (*prunus mume*), and cabbage (*Brassica rapa* L.) [12, 14–17]. The expression levels of most human lncRNA genes are lower than those of protein-coding genes, and lncRNA genes are not conserved even for closely related species [18]. In recent years, a number of lncRNAs have been reported to involve in diverse plant biological processes, such as plant growth, flowering, anthocyanin biosynthesis, and biotic/abiotic stress. For example, a lncRNA *salicylic acid biogenesis controller 1* (*SABC1*) functions as a molecular switch to balance plant defense and growth in Arabidopsis [19]. In healthy plants, *SABC1* represses the expression of its neighboring gene *NAC3* via H3K27me3, which further inhibits the transcription of the *isochorismate synthase 1* (*ICS1*) responsible for salicylic acid (SA) biosynthesis. Conversely, the repression by *SABC1* was released to enhance plant resistance to pathogen infection [19]. Moreover, the lncRNA *AUXIN REGULATED PROMOTER LOOP* (*APOLO*) interacts with the transcription factor WRKY42 to control the transcription of *ROOT HAIR DEFECTIVE 6* (*RHD6*), ultimately leading to the root-hair elongation [20]. In apples, the lncRNA *MdLNC610* positively regulates high light-induced anthocyanin accumulation through modulating the expression of *MdACO1* and inducing the ethylene production [21]. Furthermore, two lncRNAs, cold-induced antisense intragenic RNA (*COOLAIR*) and cold-assisted intronic non-coding RNA (*COLDAIR*), are identified to promote flowering in Arabidopsis by silencing the floral repressor *Flowering Locus C* (*FLC*) [22, 23]. Interestingly, some lncRNAs act as endogenous target mimics (eTM) to interrupt the binding of microRNAs (miRNAs) to their targets [24]. For example, overexpression of the lncRNA *TCONS_00021861* confers tolerance to drought stress in rice by means of attenuating the repression of *miR528-3p* to the expression of critical auxin biosynthesis gene *YUCCA7* [25]. These findings clearly demonstrated that lncRNAs play a vital regulatory role in plant growth and secondary metabolism.

However, lncRNAs in ginger are not yet identified so far, and the relationship between lncRNAs and gingerol biosynthesis remains still unknown. Recently, a study based on integrative transcriptome and phytochemical analyses has unraveled the critical lncRNAs modulating secondary metabolites in Oolong tea [26]. Accordingly, in this study, we firstly characterized metabolites in the mature rhizomes of three main ginger cultivars in China: leshanhuangjiang (lshj), tonglingbaij (tlbj), and yujiang 1 hao (yj1h). To identify ginger lncRNAs and to investigate the regulatory role of them in gingerol biosynthesis, genome-wide high-throughput sequencing was performed. Subsequently, lncRNAs involved in gingerol biosynthesis were tentatively identified and characterized through integrating analysis of transcriptome and metabolome. Finally, a putative regulatory network of lncRNAs, GBEGs, and TF genes, were proposed. The results not only highlight novel insights into the understanding of the gingerol biosynthesis in ginger, but also lay a basis for functional research of the candidate lncRNAs.

## Results

### Metabolites in the rhizomes of three ginger cultivars

**Fig. 1:**
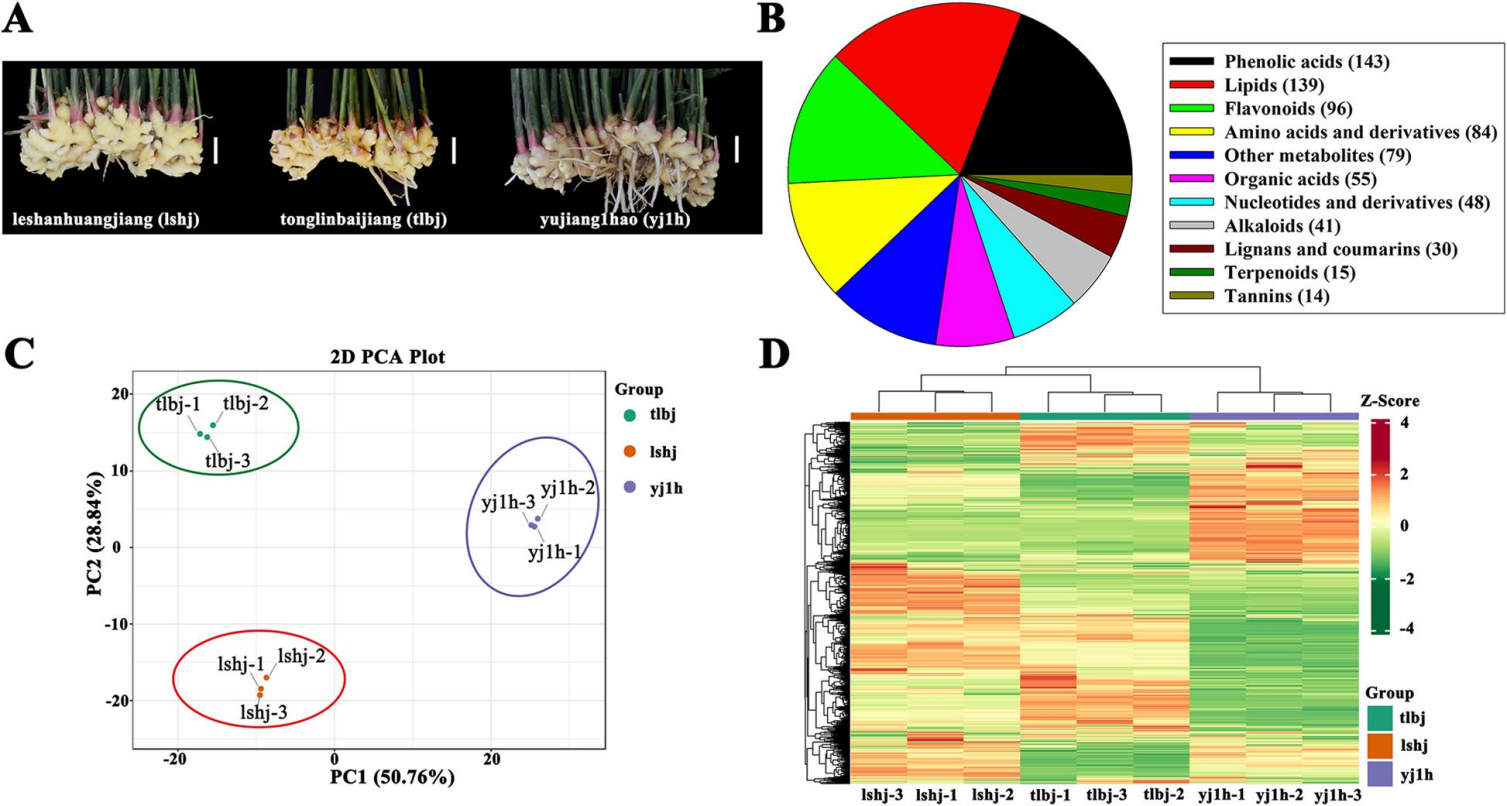
**A** The phenotypes of mature rhizomes of the three ginger cultivars. Scale bar = 5 cm. **B** The pie chart of 744 metabolites. **C** The PCA analysis of metabolite data. The x-axis represents principal component 1 (PC1); the y-axis represents principal component 2 (PC2); the three ginger varieties are distinguished by different colors. **D** HCA analysis based on the relative content of metabolites in the rhizomes of three ginger varieties

In total, 744 metabolites were identified by an ultra-performance liquid chromatography-electrospray ionization tandem triple quadrupole /mass spectrometry (UPLC-ESI–MS/MS), which were divided into eleven categories (Fig. 1B, Fig. S1, Table S2). Among them, the phenolic acid category contained 143 metabolites, which was the largest group, followed by lipids, flavonoids, amino acids and derivatives, others, organic acids, nucleotides and derivatives, alkaloids, lignans and coumarins, terpenoids, and tannins (Fig. 1B). The amounts of [6]-gingerol, [8]-gingerdione, [6]-shogaol, [10]-shogaol, and [8]-gingerol were the top five compounds in phenolic acid category (Table S2). Moreover, lysoPC 18:2(2n isomer), diosmetin-7-O-rutinoside (diosmin), L-isoleucine, 5-hydroxy-1,7-bis(4-hydroxy-3-methoxyphenyl)heptan-3-one, citric acid, adenosine, spermine, 7-methoxy-5-prenyloxycoumarin, nootkatol, and procyanidin B3, were the highest compound in other ten metabolic categories, respectively (Table S2). These results imply that ginger rhizomes are rich in various bioactive compounds, especially phenolic acids.

Next, these metabolites were analyzed by principal component analysis (PCA). The results showed that lower variability was found among the three biological replicates for each ginger cultivar (Fig. 1C). Principal component 1 and principal component 2 accounted for 50.76% and 28.84% of the metabolic variance among these samples, respectively, and resulting in a distinct separation of three cultivars. Furthermore, hierarchical cluster analysis (HCA) was conducted to detect changes in metabolites in all samples based on their relative contents (Fig. 1D). Consistent with the results of the PCA analysis, the HCA results showed that the contents of most metabolites detected in the rhizomes had high heterogeneity between different ginger cultivars, while there was high homogeneity among the three biological replicates of each variety. Furthermore, the correlation among all metabolite data was analyzed, as shown in Fig. S2. These results suggest that there was a good correlation among replicates.

### The metabolic diversity in the rhizomes of three ginger cultivars

**Fig. 2:**
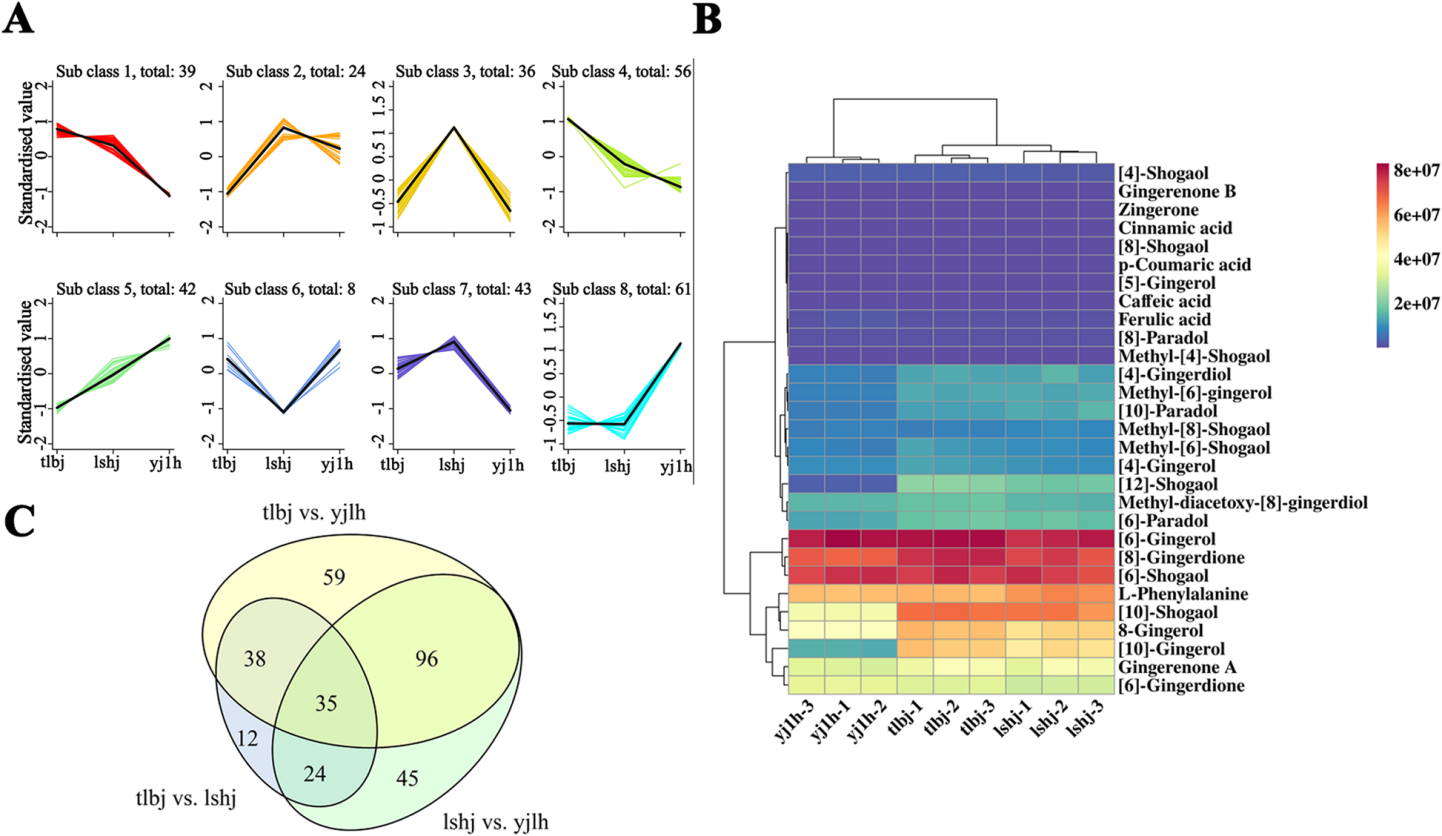
**A** K-means clustering groups of the differentially accumulated metabolites of the three ginger varieties. The y-axis represents the standardized content per metabolite, and the x-axis represents the different varieties. **B** The heat map of the content of 24 gingerol analogs and 5 biosynthetic substrates of gingerols in three ginger varieties. Different colors represent different contents, blue represents the lower contents, and red represents the higher contents. **C** Venn diagram of the differentially accumulated metabolites shared among two or three comparisons

The contents of 200 metabolites were differentially accumulated between leshanhuangjiang (lshj) and yujiang 1 hao (yj1h); 109 metabolites showed diverse enrichment in tonglingbaij (tlbj) vs. leshanhuangjiang (lshj); and 228 metabolites exhibited different changes in tonglingbaij (tlbj) vs. yujiang 1 hao (yj1h) (Fig. 2C, Tables S3-S5). Of them, 35 metabolites showed altered accumulation among all pairwise comparisons, including one amino acid and its derivative, fifteen flavonoids, two lignans and coumarins, five lipids, one nucleotide, and its derivative, four organic acids, three phenolic acids, one tannin, and three other metabolites (Fig. 2C). Additionally, based on K-means analysis, all metabolites with diverse accumulation in three cultivars could be divided into eight subclasses (Fig. 2A). In these subclasses, cultivar-specific subclasses were identified. For instance, tlbj was rich in metabolites in subclasses 1 and 4; and lshj was rich in metabolites in subclasses 2, 3, and 7. However, the metabolites in subclasses 5 and 8 were the richest in yj1h (Fig. 2A).

As for the importance of gingerols in ginger, we specifically compared the contents of the gingerols and gingerol biosynthetic substrates in this study. Noticeably, a total of 24 gingerol analogs were identified in these metabolites (Fig. 2B). Of which, 21 gingerol analogs were identified in the phenolic acid category and the other three were monitored in the others group (Table S2). Moreover, five gingerol biosynthetic substrates (L-phenylalanine, cinnamic acid, p-coumaric acid, caffeic acid, and ferulic acid), were also identified (Fig. 2B, Table S2). Notably, three derivatives of gingerols were clearly different. The [8]-shogaol (*p* < 0.001), [10]-gingerol (*p* < 0.001), and [12]-shogaol (*p* < 0.001) contents in the rhizomes of lshj were significantly higher than those in yj1h (Fig. 2B, Table S3). Similarly, the amounts of [12]-shogaol in tlbj were markedly higher (*p* < 0.001) than those in yj1h (Fig. 2B, Table S5). Additionally, compared to lshj, the accumulation of [8]-shogaol was significantly lower (*p* < 0.001) in tlbj (Fig. 2B, Table S4).

### High-throughput sequencing and genome-wide identification of lncRNAs in ginger

**Fig. 3:**
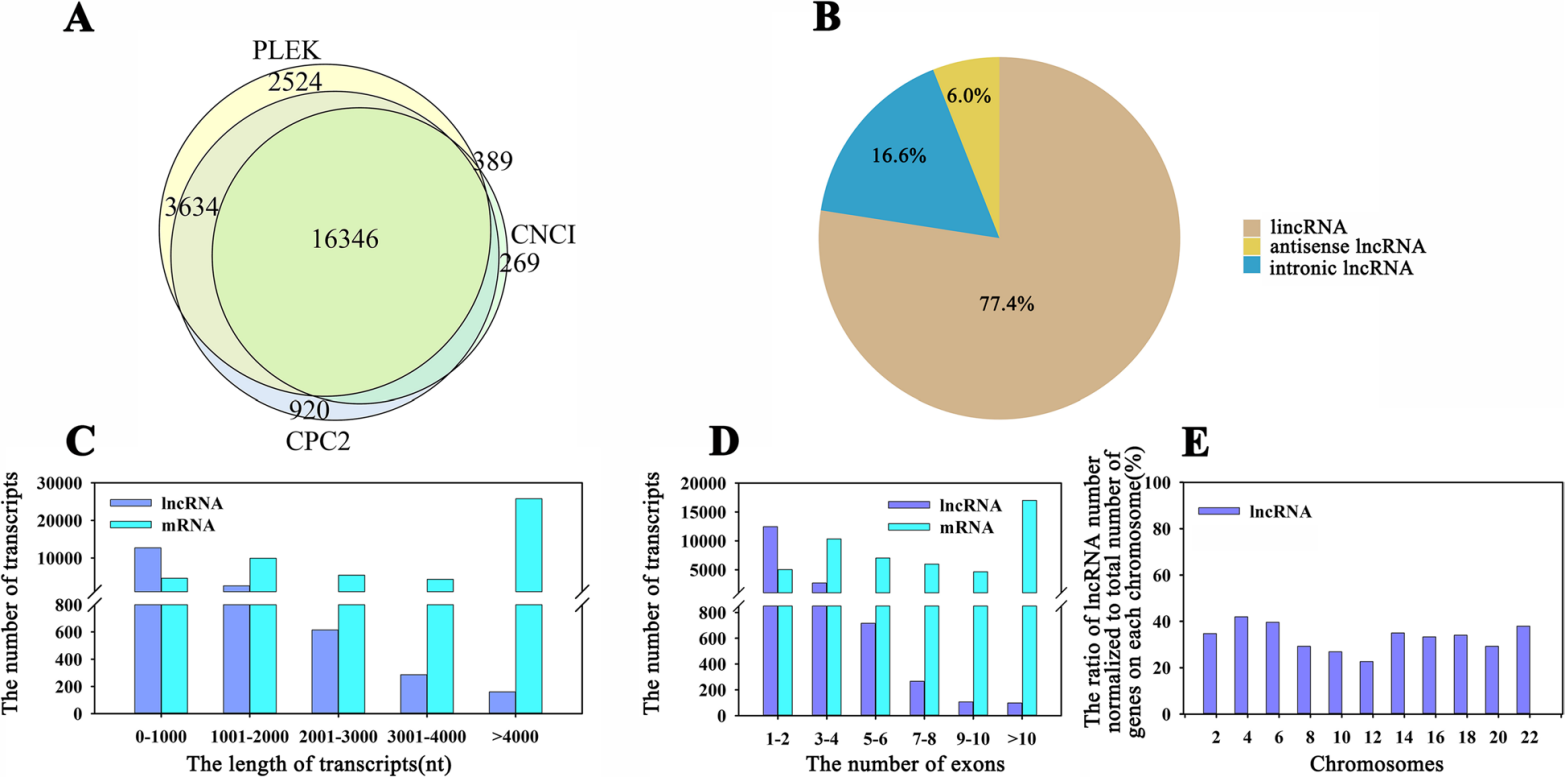
Identification and characterization of lncRNAs and protein-coding genes in three ginger varieties. **A** Venn diagram analysis of identified lncRNAs using CPC2, CNCI, and PLEK software. **B** The proportions of different types of lncRNAs. **C** Comparison of transcript length between lncRNAs and protein-coding genes. **D** Comparison of the exon number between lncRNAs and protein-coding genes. **E** Genomic locations of all the lncRNAs and protein-coding genes

Totally, 93,500,930, 87,662,184, 88,946,816, 81,385,016, 95,576,162, 93,040,956, 185,002,632, 89,949,264 and 92,583,882 raw reads were gained from 9 libraries, respectively. Correspondingly, 92,361,278, 86,356,308, 87,596,080, 80,088,006, 94,078,706, 91,776,760, 181,826,106, 88,852,328, and 91,640,532 clean reads with high Q20 and Q30 were obtained from each library, respectively (Table S6). Of the 69.60%–80.88% clean reads which could be mapped to our *Z. officinale* Roscoe genome, 56.80%–64.13% of them were uniquely mapped reads (Table S7). In total, 16,346 lncRNAs were identified (Fig. 3A, Tables S8). According to the genomic location relationship between lncRNAs and known protein-coding genes, these lncRNAs were divided into three categories: 12,661 (77.4%) long intergenic lncRNAs (lincRNAs, that do not overlap with the exons of any other genes), 978 (6.0%) antisense lncRNAs (those are located on the opposite strand of a known protein-coding gene and transcribed in the antisense direction), and 2,707 (16.6%) intronic lncRNAs (that are located and transcribed within introns of known protein-coding genes) (Fig. 3B). Moreover, the length, exon number, and chromosome distribution of the lncRNAs were compared with those of the protein-coding genes. The results showed that the length of most lncRNAs was 201–1,000 nt, and the number of lncRNAs gradually decreased along with increasing transcript length. Furthermore, the length of the lncRNAs ranged from 201 to 11,020 bp, with an average length of 789 bp (Table S8). Interestingly, protein-coding genes over 4,000 nt in length were higher in number in our RNA-seq data (Fig. 3C). Furthermore, most lncRNAs had fewer than six exons, whereas more than 15,000 protein-coding genes possessed more than 10 exons (Fig. 3D). Additionally, these lncRNAs were distributed on 11 chromosomes, with the largest numbers on chromosomes 4 and 6, respectively (Fig. 3E). It also showed that the expression levels of lncRNAs were lower than those of protein-coding genes (Fig. S3).

### Identification of differentially expressed lncRNA genes (DELs) and protein-coding genes (DEPCGs)

To screen for differentially expressed (DE) lncRNA genes and protein-coding genes in the three ginger cultivars, three pairwise comparisons were analyzed in this study. Based on the threshold value of |log2(fold change)|> 1 and an adjusted *p*-value < 0.05, 2,513 (2,013 upregulated and 510 downregulated), 1,225 (791 upregulated and 434 downregulated), and 2,884 (2,520 upregulated and 364 downregulated) DELs were identified in the comparisons between lshj vs. yj1h, tlbj vs. lshj, and tlbj vs. yj1h, respectively (Fig. S4A-C, Table S9-S11). Venn analysis revealed that 129 DELs exhibited transcriptional changes in all three comparisons (Fig. S4D). In addition, 3,898 (2,339 upregulated and 1,559 downregulated), 2,281 (1,104 upregulated and 1,177 downregulated), and 4,221 (2,613 upregulated and 1,608 downregulated) DEPCGs were identified in the comparisons of lshj vs. yj1h, tlbj vs. lshj, and tlbj vs. yj1h, respectively (Fig. S5A-C, Table S12-S14). Of which, 181 DEPCGs were shared in all three comparisons via the Venn analysis (Fig. S5D). Additionally, the K-means analysis showed that these DELs were divided into four types of expression patterns in the three ginger cultivars. The expression levels of DELs in subclasses 1 and 4 were the highest in the rhizomes of yj1h. Conversely, the DELs transcripts in subclasses 2 and 3 were higher in lshj and tlbj, respectively (Fig. S6A). Noticeably, the K-means analysis exhibited that these DEPCGs were divided into ten types of different expression patterns. For example, the DEPCGs expression levels in subclasses 1, 4, 7, and 8 were higher in yj1h, while the transcripts of DEPCGs in subclasses 6, 9, and 10 were higher in tlbj; conversely, the DEPCGs in subclasses 2, 3, and 5 showed higher expression levels in lshj (Fig. S6B).

### Analysis of potential DEPCG targets of DELs

**Fig. 4:**
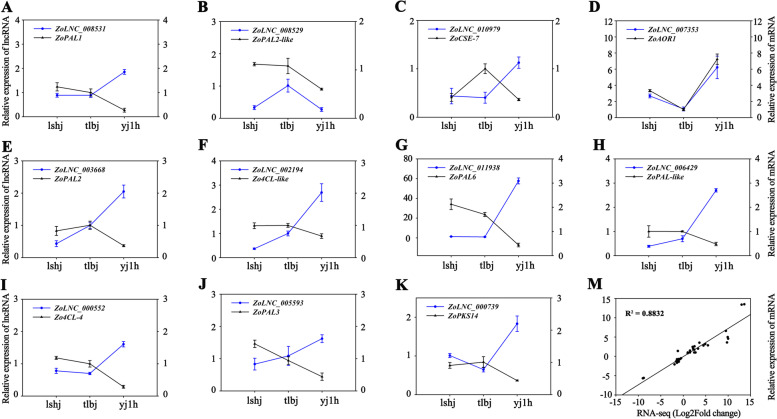
The relative expression levels of 11 gingerol biosynthesis genes and 11 corresponding lncRNAs in three ginger varieties by qRT-PCR. The values represent the means ± SD (*n* = 3) with three biological replicates

DEPCG targets located within 100 kb upstream or downstream of DELs were screened as potential *cis*-regulated targets of DELs [27]. 883 out of 2,513, 350 out of 1,225, and 1,123 out of 2,884 DELs identified potential *cis*-regulated targets in lshj vs. yj1h, tlbj vs. lshj, and tlbj vs. yj1h, respectively. Several DELs possessed at least two DEPCG targets (Fig. S7A, Table S15-S17). In addition, we screened DEPCGs showing similar expression patterns to those of DELs, which were defined as potential *trans*-regulated targets of DELs [28]. 1,437 out of 2,513, 1,222 out of 1,225, and 1,358 out of 2,884 might play a *trans*-acting role in the regulation of DEPCGs. Similarly, those DELs might function as common upstream regulators to abundant DEPCGs (Fig. S7B, Table S18). Next, to validate the results of potential DEPCG targets of DELs, qRT-PCR was performed. The expression patterns of 11 DEPCGs and 11 corresponding DELs were monitored. Among them, *ZoLNC_008531*, *ZoLNC_008529*, *ZoLNC_010979*, and *ZoLNC_007353* regulated the expression levels of *ZoPAL1*, *ZoPAL2-lik*e, *ZoCSE-7*, and *ZoAOR1*, respectively, in the *cis*-acting model. The *ZoLNC_003688*, *ZoLNC_002194*, *ZoLNC_011938*, *ZoLNC_006429*, *ZoLNC_000552*, *ZoLNC_005593*, as well as *ZoLNC_000739* responded to control the transcripts of *ZoPAL2*, *Zo4CL-like*, *ZoPAL6*, *ZoPAL-like*, *Zo4CL-4*, *ZoPAL3*, and *ZoPKS14* in the *trans*-acting model. The expression patterns of the selected DELs and DEPCGs determined by qRT-PCR were consistent with those in the RNA-seq data (R^2^ = 0.8832) (Fig. 4, Fig. S8). This suggested that the results of potential DEPCG targets of DELs were highly reliable.

### Functional analysis of DELs based on their potential targets

Based on the *cis*- and *trans*-acting models, the functions of the DELs were first analyzed through Gene Ontology (GO) enrichment analysis in the three comparisons. For instance, the DELs involved in the secondary metabolite biosynthetic process (GO:0044550), response to plant hormones (GO:0009753, GO:0010337), and phenol-containing compound metabolic process (GO:0018958), etc., were enriched (Fig. S9).

**Fig. 5:**
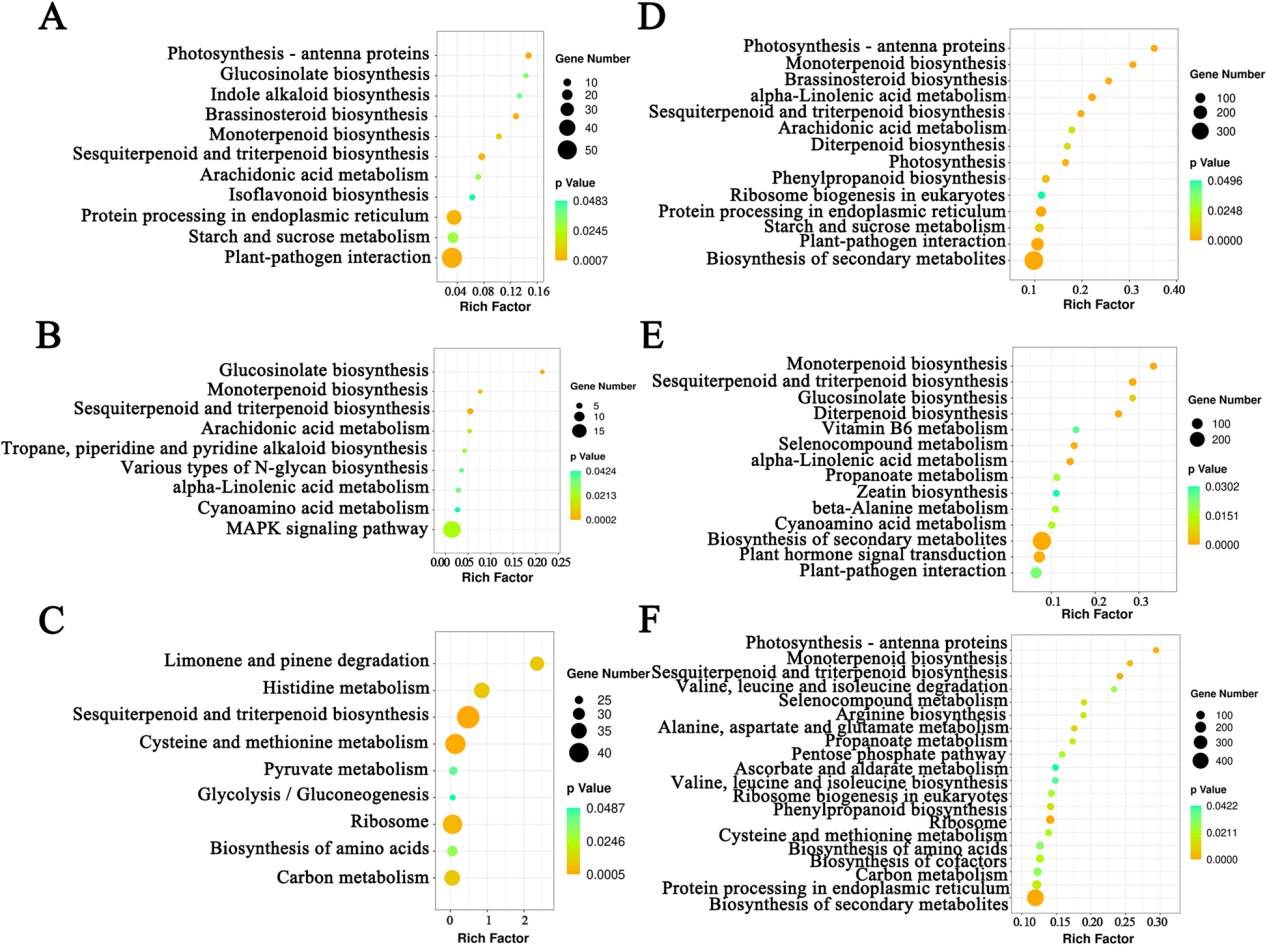
Statistical analysis of KEGG pathway enrichment of DELs based on the *cis*-acting model in the three comparisons of lshj vs. yj1h (**A**), tlbj vs. lshj (**B**), and tlbj vs. yj1h (**C**); or based on the *trans*-acting model in the three comparisons of lshj vs. yj1h (**D**), tlbj vs. lshj (**E**), and tlbj vs. yj1h (**F**). The color and the size of the circle represent the *p*-value and the number of DELs enriched in each pathway, respectively. Rich factor represents the ratio of the number of differentially expressed genes in one KEGG pathway to the total number of the genes detected in this pathway. KEGG is developed by Kanehisa Laboratories (https://www.kegg.jp/kegg/kegg1.html)

In addition, functional prediction of the DELs was performed using the Kyoto Encyclopedia of Genes and Genomes (KEGG) pathway analyses (Fig. 5). The DELs functioned in the plant hormone signal transduction (ko04075), biosynthesis of secondary metabolites (ko01110), phenylpropanoid biosynthesis (ko00940), MAPK signaling pathway (ko04016), etc., were enriched (Fig. 5).

### The contributions of DELs to gingerol biosynthesis by targeting the key enzyme genes and transcription factor genes in ginger rhizomes

**Fig. 6:**
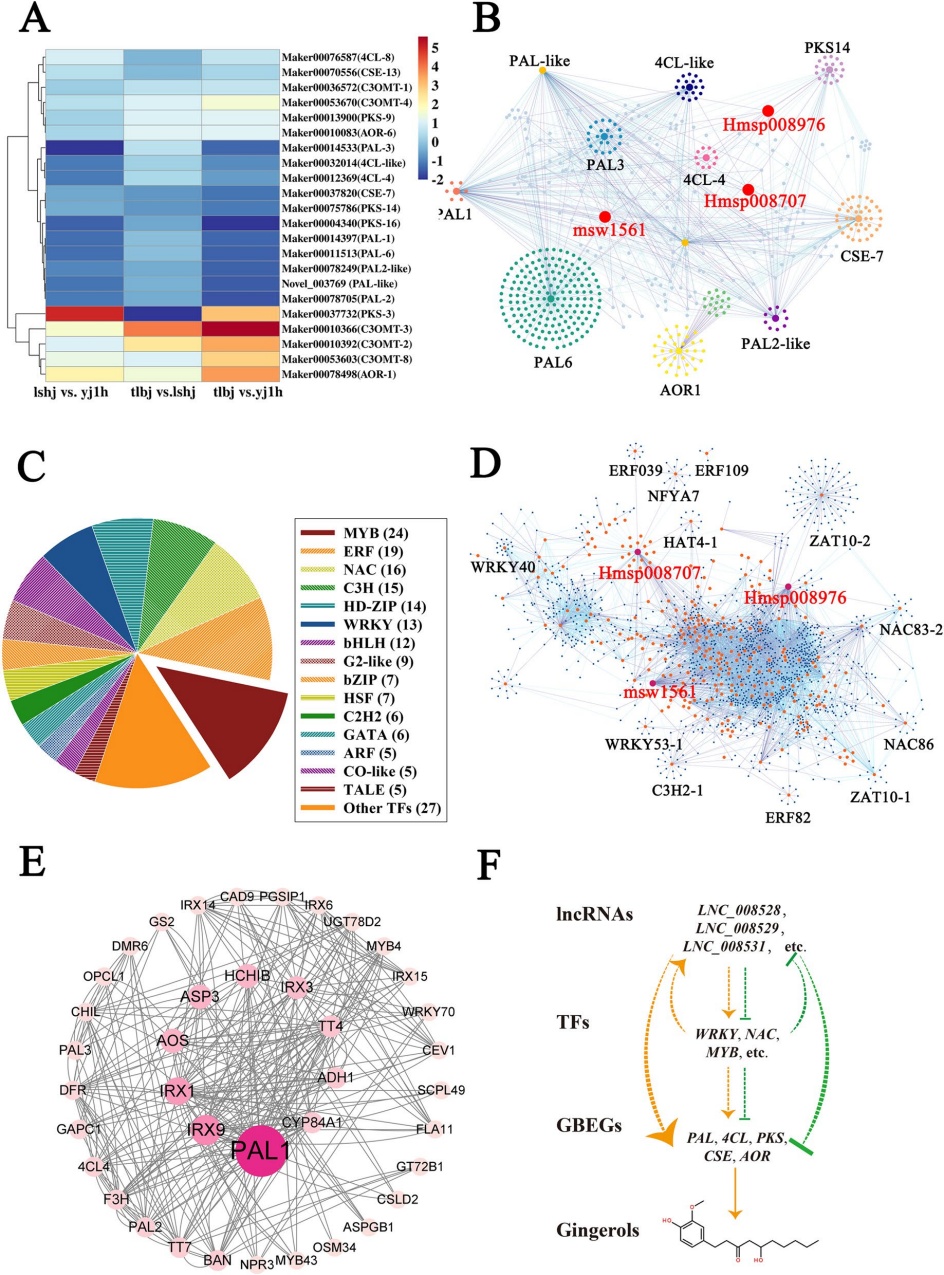
**A** The heat map of the expression levels of 22 differentially expressed gingerol biosynthesis enzyme genes. Different colors represent different expression levels, blue represents the lower expression levels, and red represents higher transcripts. **B** Co-expression network of 3 gingerol analogs ([10]-gingerol (mws1561), [12]-shogaol (Hmsp008976), and [8]-shogaol (Hmsp008707)), 11 gingerol biosynthetic genes as well as their corresponding 1,200 *trans*-acting lncRNAs. The different marker size was used to represent metabolites, GBEGs and lncRNAs, respectively. The largest markers represent metabolites, medium markers represent genes, and small markers represent lncRNAs. **C** The pie chart of 190 TF genes showing a high correlation coefficient with 3 gingerol analogs. The count of TF genes belonging to each TF families are given in parenthesis **D** Co-expression network of 3 gingerol analogs ([10]-gingerol (mws1561), [12]-shogaol (Hmsp008976), and [8]-shogaol (Hmsp008707)), 190 TF genes as well as their corresponding 2,225 *trans*-acting lncRNAs. The different marker size was used to represent metabolites, TF genes and lncRNAs, respectively. The largest markers, medium markers, as well as small markers represent metabolites, genes, and lncRNAs, respectively. **E** Protein–protein interaction networks (PPI) of 4 GBEGs proteins (PAL1, PAL2, PAL3, and 4CL-4) and their potential 44 interaction proteins. **F** The three-tier regulatory model for lncRNAs regulation of gingerol biosynthesis in ginger. lncRNAs could regulate the biosynthesis of gingerols via directly or indirectly modulating the GBEGs expression under *cis*/*trans*-acting models. Furthermore, lncRNAs could directly or indirectly control the transcripts of TFs genes under *cis*/*trans*-acting models, which then directly or indirectly modulate GBEGs expression to regulate gingerol biosynthesis. Meanwhile, these TFs also could directly or indirectly feedback control lncRNAs expression

To obtain the DELs that participated in the regulation of gingerol biosynthesis, we first screened differentially expressed (DE) gingerol biosynthesis enzyme genes (GBEGs) in the three comparisons. Totally, 22 DE GBEGs were identified (Fig. 6A). Subsequently, a correlation analysis was constructed between three significantly different gingerols ([10]-gingerol (mws1561), [12]-shogaol (Hmsp008976), and [8]-shogaol (Hmsp008707)) and 22 DE GBEGs (Table S19). Interestingly, the expression levels of 11 DE GBEGs (*PAL-like*, *PAL1*, *PAL2*, *PAL2-like*, *PAL3*, *PAL6*, *AOR1*, *4CL-4*, *4CL-like*, *CSE7*, and *PKS14*) showed a high correlation coefficient with the contents of three gingerol analogs (*p*-value < 0.01), Table S20). Moreover, DELs with a potential role in controlling the 11 GBEGs were screened. Among them, *PAL1*, *PAL2-like*, *PAL3*, *CES-7*, and *AOR1* could be targeted by 7 DELs using a *cis*-acting model (Table S21). Using a *trans*-acting model, 1,200 DELs were identified as upstream regulators to the 11 GBEGs (Fig. 6B, Table S22). Interestingly, some DELs may target several gingerol biosynthesis enzyme genes. For example, three DELs (*LNC_014041*, *LNC_008010*, and *LNC_005460*) have been identified as potential regulators of at least seven gingerol biosynthetic enzyme genes (Fig. 6B, Table S21, S22).

Next, we screened the differentially expressed transcription factor (DE TF) genes, which expression patterns shown a high correlation coefficient with the contents of three gingerol analogs in three ginger cultivars (*p*-value < 0.01). A total of 190 DE TF genes were identified, which were grouped into 32 TF families (Fig. 6C, Table S23). Among them, 24 TF genes belonged to MYB TF family, which were the largest group, followed by ERF TF family (19 members), NAC TF family (16 members), C3H TF family (15 members), HD-ZIP TF family (14 members), etc. (Fig. 6C, Table S23). *Cis*-acting model analysis indicated that 83 out of 190 TF genes possessed their corresponding 111 upstream *cis*-regulating DELs (Table S24). Interestingly, all 190 TF genes could be targeted by 2,225 *trans*-regulating DELs (Fig. 6D, Table S25). Moreover, we analyzed the potential interaction proteins to 11 GBEGs by protein–protein interaction networks (PPI) analysis. 44 proteins were identified to interact with 4 GBEGs including PAL1, PAL2, PAL3, and 4CL4. Among them, 3 TF proteins (MYB4, MYB43, and WRKY70) and 2 flavonoid biosynthesis related proteins (TT4 and TT7) were found (Fig. 6E). Based on these results, a network of multiple critical lncRNAs in gingerol biosynthesis was constructed (Fig. 6F). However, further studies are needed to better understand their molecular mechanisms.

## Discussion

In the present study, a total of 744 metabolites were identified in the ginger rhizomes using an ultra-performance liquid chromatography-electrospray ionization tandem triple quadrupole/mass spectrometry (UPLC-ESI–MS/MS), including 24 gingerol derivatives and 5 biosynthetic substrates of gingerols (Figs. 1 and 2, Table S2). The number of metabolites was higher than that measured by an ultra-performance liquid chromatography-electrospray ionization tandem triple quadrupole/mass spectrometry (UPLC-MS/MS) analysis [1]. This suggests that UPLC-ESI–MS/MS is a powerful tool for detecting metabolites in ginger; and a similar effect was observed in buckwheat [29]. Additionally, these differentially accumulated metabolites could be divided into eight subclasses, and the cultivar-specific subclasses were identified using K-means analysis (Fig. 2A). These results could not only help us to better evaluate the edible and medicinal value of the three ginger cultivars, but also provide a theoretical basis to manipulate different gingerol content in ginger breeding.

Noticeably, the contents of [10]-gingerol, [12]-shogaol, and [8]-shogaol were significantly different among the three cultivars (Fig. 2, Tables S3-S5). The concentrations of [10]-gingerol and [12]-shogaol in the rhizomes were in the order tonglingbaijiang (tlbj) > leshanhuangjiang (lshj) > yujiang1hao (yj1h) (Fig. 2, Table S2). The accumulation of [8]-shogaol was higher in lshj than in tlbj and yj1h, whereas no significant difference was found between tlbj and yj1h (Fig. 2, Table S2). This is consistent with our previous report that the yj1h is more suitable for use as a table vegetable because of its lower gingerol content [30].

Moreover, a total of 16,346 lncRNA genes were identified in ginger by RNA-seq, including 12,661 (77.4%) long intergenic lncRNAs (lincRNAs), 978 (6.0%) antisense lncRNAs, and 2,707 (16.6%) intronic lncRNAs (Fig. 3). The results showed that ginger contains a higher number of lincRNAs than other plant species, such as maize and rice [16, 31]. Furthermore, the structural analysis revealed that the average length of lncRNA genes was shorter than that of protein-coding genes, and the expression levels of lncRNA genes were lower than those of protein-coding genes (Fig. 3C, Fig. S3), which are in agreement with those of previous studies [32]. Interestingly, the mean length (789 bp) of the lncRNAs in ginger was longer than that of other species, that is, 285, 364, 463, and 323 bp for Arabidopsis, maize, kiwifruit, and rice, respectively. It is because the average length of lncRNAs is different in species [33].

Additionally, 2,513, 1,225, and 2,884 differentially expressed lncRNAs (DELs) were identified in the comparisons of lshj vs. yj1h, tlbj vs. lshj, and tlbj vs. yj1h, respectively (Fig. S4, Table S9-S11). Among them, 7 *cis*-acting and 1,200 *trans*-acting DELs were respectively identified as upstream regulators to 11 gingerol biosynthesis enzyme genes (GBEGs), which showed a highly significant correlation coefficient (*p*-value < 0.01) with three differentially accumulated ginger analogs (Fig. S7, Table S21 and S22). In particular, *4CL* is a critical gene for catalyzing steps involving p-coumaroyl-CoA and feruloyl-CoA, thereby generating enough substrates for the synthesis of gingerols [1, 34]. In the present study, we found that the transcripts of two *4CL* genes (*4CL-4* and *4CL-like*) showed a strongly positive correlation of expression levels with the contents of [10]-gingerol (mws1561, index = 0.937, *p*-value = 0.00018) and [12]-shogaol (Hmsp008976, index = 0.931, *p*-value = 0.00027) in ginger rhizomes and might be targeted by 512 and 352 *trans*-acting DELs, respectively (Fig. 6A and B, Tables S21 and S22). These data provide evidence that lncRNAs are related to ginger analogs biosynthesis via the regulation of various GBEGs, specifically *4CL* expression. In oolong tea, *4CL* expression was inhibited by the low expression of lncRNA *LTCONS-00054003*, which participates in the regulation of flavonoid accumulation [26]. Interestingly, the DE *C3OMT* family gene, *C3OMT-2*, its expression levels did not exhibit a high correlation coefficient to the contents of [8]-shogaol (index = 0.027, *p*-value = 0.944) in our research, which has been identified as a critical gene that participated in gingerols biosynthesis in previous study [1].

On the other hand, we also found that 32 transcription factor (TF) families including 190 TF genes had a highly significant correlation coefficient (*p* value < 0.01) with the amounts of [8]-shogaol, [10]-gingerol, or [12]-shogaol. (Fig. 6C, Table S23). These TF genes were significantly enriched in all DEGs by 2 × 2 Fisher exact test (*p*-value = 1.433e-13). Furthermore, among these TF proteins, MYB4, MYB43, and WRKY70 might interact with PAL1, PAL2, PAL3, and 4CL4 (Fig. 6E). These results suggested that TFs genes might participate in gingerol biosynthesis not only through regulation of GBEGs gene expression at transcriptional level, but also by affecting GBEGs enzyme activities at post-transcriptional level. We also found that 111 *cis*-acting and 2,225 *trans*-acting DELs could regulate these TF genes expression levels, suggesting that lncRNAs also participate in controlling gingerol biosynthesis by regulating the transcription of TFs genes (Fig. 6D, Tables S24 and S25). Similar results have demonstrated that the lncRNA/TF regulatory module controls other secondary metabolic biosynthesis, such as anthocyanin biosynthesis. For example, in sea buckhorn fruits, one lncRNA *LNC1* promotes anthocyanin biosynthesis by acting as endogenous target mimics of *miR156a* to reduce the expression levels of *SPL9*; in contrast, *LNC2* reduces anthocyanin biosynthesis by inducing transcripts of *MYB114* [32]. Moreover, it was recently reported that the MdWRKY1-MdLNC499-MdERF109 transcriptional cascade is involved in light-induced anthocyanin accumulation in apples [35].

In addition to controlling gingerol biosynthesis, we also found DELs involved in other biological processes, such as plant hormone signal transduction (ko04075), plant–pathogen interactions (ko04626), and MAPK signaling pathway (ko04016) (Fig. 5). It is supported by the previous conclusions [34]. For example, in apples, the lncRNA *MdLNC610* positively regulates high light-induced anthocyanin accumulation by modulating the expression of *MdACO1* and inducing ethylene production [21]. This is also consistent with kiwifruits in response to *Psa* infection [36]. The related transgenic evidence was reported in Chinese cabbage, and when a long noncoding natural antisense transcript of the *MAPK* gene (*MSTRG.19915)* was silenced, the resistance to downy mildew was enhanced [12].

## Conclusion

In this study, 200, 109, and 228 differentially accumulated metabolites were identified between ginger cultivars leshanhuangjiang (lshj) and yujiang1hao (yj1h), tonglingbaijiang (tlbj) and leshanhuangjiang (lshj), and tonglingbaijiang (tlbj) and yujiang1hao (yj1h), respectively. Among them, the concentrations of three gingerol analogs, [8]-shogaol, [10]-gingerol, and [12]-shogaol, accumulated significantly. Moreover, the expression levels of 11 gingerol biosynthesis enzyme genes and 190 transcription factor (TF) genes were highly correlated with the content of the three gingerol analogs. Additionally, 16,346 lncRNAs were identified, of which 2,513, 1,225, and 2,884 were differentially expressed in the three comparisons, respectively. Noticeably, DE lncRNAs, such as *ZoLNC_008531*, *ZoLNC_008529*, and *ZoLNC_007353*, might contribute to gingerol biosynthesis by controlling the key enzyme genes or TF transcripts via a *cis*-acting or *trans*-acting model. Overall, our results not only provide novel insight into gingerol metabolism, but also lay a foundation for future in-depth studies of the related molecular mechanism.

## Materials and methods

### Plant materials

Three ginger cultivars, leshanhuangjiang (lshj), tonglingbaijiang (tlbj), and yujiang1hao (yj1h), were used as the materials in this study. These ginger plants were planted in the greenhouse at Chongqing University of Sciences and Arts, Yongchuan, Chongqing, China (29°14’N, 105°52’E) in 2020. The growth conditions were set at 25 ± 3 °C with 14 h/10 h light/dark cycles and 60 ± 5% relative humidity. For metabolomics and transcriptomic analyses, ginger plants were randomly harvested after 180 d of growth (Fig. 1A) [1, 2]. Three biological replicates of each cultivar were collected, and each replicate consisted of pooled five mature rhizomes of each ginger cultivar. After rinsing with Milli-Q water, the rhizomes were immediately frozen in liquid nitrogen and stored at -80 °C until subsequent analyses.

### Metabolite extraction and ultra-performance liquid chromatography-electrospray ionization tandem triple quadrupole/mass spectrometry analysis

Sample preparation and extraction were performed according to the methods described by Metware Biotechnology Co., Ltd. (Wuhan, China) [29]. Briefly, the five rhizomes of each ginger cultivar were pooled and used as one biological replicate. Three biological replicates were used for each cultivar. For metabolite extraction, the samples were first desiccated using vacuum freeze-drying. The dried samples were then crushed into a powder using a mixer mill (MM 400, Retsch) at 30 Hz for 1.5 min. One hundred milligrams of powder were dissolved in 1.2 mL of 70% aqueous methanol. In order to improve the extraction rate, the solution was vortexed for 30 s and repeated six times, and then stored at 4 ℃ overnight. The mixtures were then centrifuged at 12,000 rpm for 10 min, and the supernatant was filtered using a microporous member (0.22 μm).

Next the extracts of each sample were analyzed using an ultra-performance liquid chromatography-electrospray ionization tandem triple quadrupole/mass spectrometry (UPLC-ESI–MS/MS) system (UPLC, SHIMADZU Nexera X2, https://www.shimadzu.com.cn/; MS, Applied Biosystems 4500 QTRAP, http://www.appliedbiosystems.com.cn/). Parameters of UPLC were follows: 1) column: agilent SB-C 1.8μm, 2.1 mm × 100 mm; 2) flow rate: 0.35 mL/min. The metabolites were detected under quantitative monitoring mode by multiple reaction monitoring (MRM) according to the previous method [37]. Parameters of ESI–MS were as follows: 1) temperature: 550 ℃; 2) voltage: 5500 v (positive ion mode), -4500 v (negative ion mode). All metabolite data identified in this study were listed in Table S2. Differentially accumulated metabolites were identified based on thresholds with an absolute fold change ≥ 2 or ≤ 0.5. Venn diagram of the differentially accumulated metabolites shared among two or three comparisons were analyzed on the platform of http://www.ehbio.com/test/venn/#/.

### RNA isolation, library construction, and sequencing

Total RNA was isolated from nine ginger rhizome samples using an RNeasy Plant Mini Kit (QIAGEN, Germany). The quality and integrity of the total RNA were evaluated using Nanodrop, Qubit 2.0, (Invitrogen, USA), and an Agilent Bioanalyzer 2100 System (Agilent Technologies, USA). The Epicenter Ribo-Zero™ rRNA Removal Kit (Epicenter, Madison, WI, USA) was used to remove ribosomal RNA. Three micrograms of rRNA-depleted RNA were used to construct a sequencing library using the NEBNext Ultra Directional RNA Library Prep Kit (NEB, USA) according to the manufacturer’s instructions. Nine libraries were sequenced on the Illumina PE150 platform. The sequencing results were deposited in the National Center for Biotechnology Information database under accession number (PRJNA870703), which was released since November 12^th^, 2022.

### Identification of lncRNAs and protein-coding genes

After RNA-seq, clean reads were generated by trimming the adaptor and removing low-quality reads. The clean reads were aligned to the reference genome of ginger using Hisat2 software [2, 38]. The mapped reads of each sample were assembled using the StringTie and Cuffmerge software [39]. Venn diagram of the lncRNAs shared among two or three software were analyzed on the platform of http://www.ehbio.com/test/venn/#/.

The potential lncRNAs were screened according to the follows: (1) transcripts less than 200 nt in length were removed; (2) transcripts that were known as protein-coding genes or other non-coding RNA, such as rRNA and tRNA, were also removed; (3) transcripts that possessed protein-coding ability were removed through evaluation by using coding potential calculator 2 (CPC2, CPC score > 0), coding-non-coding index (CNCI, CNCI score > 0), and predictors of long non-coding RNAs and messenger RNAs based on an improved k-mer scheme (PLEK) [40]; and (4) based on their location in the genome of the remaining transcripts, the remaining RNAs were classified into long intergenic non-coding RNAs (lincRNAs), anti-sense lncRNAs, and intronic lncRNAs [13].

### Identification of differentially expressed lncRNAs (DELs) and protein-coding genes (DEPCGs)

To determine the DELs and DEPCGs between any two cultivars, including lshj vs. yj1h, tlbj vs. lshj, and tlbj vs. yj1h, the expression levels of all lncRNAs and protein-coding genes in each sample were first quantified using fragments per kilobase per million base pairs sequenced (FPKM) values using StringTie software [41]. The transcripts of all lncRNAs and protein-coding genes in all pairwise comparisons with an adjusted *p*-value < 0.05, and an absolute fold-change value > 2.0, were defined as DELs and DEPCGs. In addition, the DELs and DEPCGs were clustered using K-means clustering with BMKCloud software (http://www.biocloud.net) [42]. Venn diagram of DELs or DEPCGs shared among two or three comparisons were analyzed on the platform of http://www.ehbio.com/test/venn/#/.

### Prediction of potential *cis*-/*trans*-targets of DELs and functional enrichment analysis of DELs

It has been reported that lncRNAs participate in the regulation of target gene expression mainly via *cis*-acting regulation (genes in close genomic proximity) or *trans*-acting regulation (genes with long distances and similar expression patterns) [27]. Thus, these DEPCGs within 100 kb upstream or downstream of the DELs were screened as *cis*-targets. Moreover, the protein-coding genes were identified as the *trans*-targets of DELs based on the correlation coefficient in expression levels between DELs and DEPCGs, with a *p*-value < 0.01 [28].

Functional enrichment analyses of DELs were performed by Gene Ontology (GO) terms and Kyoto Encyclopedia of Genes and Genomes (KEGG) pathway enrichment analyses [28, 43]. The GO ontologies were assigned using Blast2GO [28]. Among them, the GO terms (adjusted *p-*values < 0.05) were identified as significantly enriched [11]. Additionally, to understand the main pathways of DELs involved, the targets of DELs were mapped to the Kyoto Encyclopedia of Genes and Genomes (KEGG, www.kegg.jp/kegg/kegg1.html), and enrichment analysis was conducted using KOBAS 2.0 (https://www.biostars.org/p/200126/) [43]. Significantly enriched pathways were considered when *p*-values < 0.05 [14].

### Integrated analysis of the metabolites and transcriptome

To explore the association between metabolites and the transcriptome, the Pearson correlation coefficients between lncRNAs, protein-coding genes, and metabolites expression levels were calculated using the *Cor* function in R language (Metware Biotechnology Co., Ltd. Wuhan, China). Correlation analysis was performed using the quantitative values of genes and metabolites in all samples. The threshold of the correlation coefficient was set as a *p* < 0.01 [37]. Co-expression network diagrams were drawn by Cytoscape v3.8 software.

### The quantitative real-time PCR and statistical analysis

Total RNAs was isolated from nine ginger rhizome samples using a FastPure® Plant Total RNA Isolation Kit (Polysaccharides & Polyphenolics-rich) according to the manufacturer’s instructions (Cat. RC401-01, Vazyme, Nanjing, China). cDNA was synthesized using the HiScritpt® III 1st Strand cDNA Synthesis Kit (+ gDNA wiper) (Cat. R312-01, Vazyme, Nanjing, China). Gene expression levels were evaluated using a Bio-Rad CFX Connect Real-Time System with iTaq Universal SYBR® Green Supermix (Cat. 1725124, Bio-Rad, USA). The reaction conditions were as follows: denaturation at 95 °C for 3 min, followed by 40 cycles of 95 °C for 15 s, 60 °C for 30 s, and 72 °C for 45 s. A melting curve analysis was then performed. The primers used are listed in Table S1, and *ZoTUB2* was used as the internal control [1, 3].

### Statistical analyses

The Student’s *t*-test was conducted to evaluate differences between two groups by SPSS Statistic 17.0. One-way ANOVA was performed to measure differences among multiple varieties, followed by a Tukey Honest Significant Differences (HSD) test for the post-hoc analysis.

### Supplementary Information

**Additional file 1:** **Fig. S1.** Integration calibration analysis of randomly selected positive (A) and negative (D) ionization compounds in different samples; x-axis represents the retention time (min) of metabolite detection, and y-axis represents the ion current intensity (cps) of ion detection. Multimodal plot analysis of positive (B) and negative (E) ionization compounds by MRM metabolite detection. Total ion chromatogram of positive (C) and negative (F) ionization compounds in mixed samples by mass spectrometry analysis.

**Additional file 2:** **Fig. S2.** Correlation analysis among the three replicates of the metabolome.

**Additional file 3:** **Fig. S3.** The expression levels of lncRNA genes (A) and protein-coding mRNA genes (B) in the rhizomes of three ginger varieties.

**Additional file 4:** **Fig. S4.** The volcano figures for the DELs in the three comparisons of lshj vs. yj1h (A), tlbj vs. lshj (B), and tlbj vs. yj1h (C), respectively. (D) Venn diagram analysis of DELs shared among two or three comparisons.

**Additional file 5:** **Fig. S5.** The volcano figures for the DEPCGs in the three comparisons of lshj vs. yj1h (A), tlbj vs. lshj (B), and tlbj vs. yj1h (C), respectively. (D) Venn diagram analysis of DEGs shared among two or three comparisons.

**Additional file 6:** **Fig. S6.** K-means clustering groups of DELs (A) and DEPCGs (B) in the three ginger cultivars. The y-axis represents the standardized value per gene, and the x-axis represents the different cultivars.

**Additional file 7:** **Fig. S7.** (A) The DEPCGs targets of DELs in three comparisons under the *cis*-acting model. (B) The DEPCGs target DELs in three comparisons under the *trans*-acting model.

**Additional file 8:** **Fig. S8.** The relative expression levels of 11 ginger biosynthesis genes and 11 corresponding lncRNAs in three ginger varieties by RNA-seq.

**Additional file 9: Fig. S9.** Statistical analysis of GO enrichment of DELs based on the *cis*-acting model in the three comparisons of lshj vs. yj1h (A), tlbj vs. lshj (B), and tlbj vs. yj1h (C), or based on the *trans*-acting model in the three comparisons of lshj vs. yj1h (D), tlbj vs. lshj (E), and tlbj vs. yj1h (F).

**Additional file 10:** **Table S1.** The primer lists. **Table S2.** All metabolic profiles in the rhizomes of three main ginger cultivars. **Table S3.** The differentially accumulated metabolites in lshj vs. yj1h. **Table S4.** The differentially accumulated metabolites in tlbj vs. lshj. **Table S5.** The differentially accumulated metabolites in tlbj vs. yj1h. **Table S6.** Statistical analysis of the RNA-seq reads for the rhizomes of three ginger varieties with three biological replicates. **Table S7.** Alignment analysis of clean reads from the nine RNA-seq libraries with the ginger genome. **Table S8.** lncRNAs information in RNA-seq data. **Table S9.** The differentially expressed lncRNAs in the lshj vs. yj1h. **Table S10.** The differentially expressed lncRNAs in the tlbj vs. lshj. **Table S11.** The differentially expressed lncRNAs in the tlbj vs. yj1h. **Table S12.** The differentially expressed mRNAs in the lshj vs. yj1h. **Table S13.** The differentially expressed mRNAs in the tlbj vs. lshj. **Table S14.** The differentially expressed mRNAs in the tlbj vs. yj1h. **Table S15.** The analysis of *cis*-acting DEPCG targets of DELs in the lshj vs. yj1h. **Table S16.** The analysis of *cis*-acting DEPCG targets of DELs in the tlbj vs. lshj. **Table S17.** The analysis of *cis*-acting DEPCG targets of DELs in the tlbj vs. yj1h. **Table S18.** The analysis of *trans*-acting DEPCG targets of DELs in the three comparisons. **Table S19.** The coefficient analysis between metabolome and RNA-seq. **Table S20.** The coefficient analysis of 11 gingerol biosynthetic enzyme genes with 3 differentially accumulated gingerol analogs. **Table S21.** These *cis*-regulatory DELs to the 5 GBEGs associated with gingerol biosynthesis. **Table S22.** These *trans*-regulatory DELs to the 11 GBEGs associated with gingerol biosynthesis. **Table S23.** The coefficient analysis of 190 TFs genes with 3 differentially accumulated gingerol analogs. **Table S24.** These *cis*-regulatory DELs to the 83 TFs genes associated with gingerol biosynthesis. **Table S25.** These *trans*-regulatory DELs to the 170 TF genes associated with gingerol biosynthesis.

## Data Availability

The raw sequence reads for the 9 sequenced libraries are available for download from the NCBI sequence read archive database (accession number and direct link: SRR21160241, https://www.ncbi.nlm.nih.gov/sra/?term=SRR21160241 (tlbj-1); SRR21160240, https://www.ncbi.nlm.nih.gov/sra/SRR21160240 (tlbj-2); SRR21160248, https://www.ncbi.nlm.nih.gov/sra/?term=SRR21160248 (tlbj-3); SRR21160249, https://www.ncbi.nlm.nih.gov/sra/?term=SRR21160249 (lshj-1); SRR21160243, https://www.ncbi.nlm.nih.gov/sra/?term=SRR21160243 (lshj-2); SRR21160242, https://www.ncbi.nlm.nih.gov/sra/?term=SRR21160242 (lshj-3); SRR21160247, https://www.ncbi.nlm.nih.gov/sra/?term=SRR21160247 (yj1h-1); SRR21160245, https://www.ncbi.nlm.nih.gov/sra/?term=SRR21160245 (yj1h-2); SRR21160244, https://www.ncbi.nlm.nih.gov/sra/?term=SRR21160244 (yj1h-3); The metabolomics raw data are available from supplementary material Table S2.

## References

[1] Li HH, Wu L, Tang N, Liu R, Jin Z, Liu YQ, Li ZG (2020). Analysis of transcriptome and phytohormone profiles reveal novel insight into ginger (Zingiber officinale Rose) in response to postharvest dehydration stress. Postharvest Biol Tec.

[2] Li HL, Wu L, Dong ZM, Jiang YS, Jiang SJ, Xing HT (2021). Haplotype-resolved genome of diploid ginger (Zingiber officinale) and its unique gingerol biosynthetic pathway. Hortic Res.

[3] Li G, Ma JW, Yin JL, Guo FL, Xi KY, Yang PH (2022). Identification of reference genes for reverse transcription-quantitative PCR analysis of ginger under abiotic stress and for postharvest biology studies. Front Plant Sci.

[4] Jiang YS, Liao QH, Zou Y, Liu YQ, Lan JB (2017). Transcriptome analysis reveals the genetic basis underlying the biosynthesis of volatile oil, gingerols, and diarylheptanoids in ginger (Zingiber officinale Rosc.). Botl Stud.

[5] Jiang YS, Huang MJ, Wisniewski M, Li HL, Zhang MX, Tao X (2018). Transcriptome analysis provides insights into gingerol biosynthesis in ginger (Zingiber officinale). Plant genome.

[6] Lee HS, Seo EY, Kang NE, Kim WK (2008). [6]-Gingerol inhibits metastasis of MDA-MB-231 human breast cancer cells. J Nutr Biochem.

[7] Semwal RB, Semwal DK, Combrinck S, Viljoen AM (2015). Gingerols and shogaols: important nutraceutical principles from ginger. Phytochemistry.

[8] Khan IA, Cao K, Guo J, Li Y, Wang Q, Yang XW (2022). Identification of key gene networks controlling anthocyanin biosynthesis in peach flower. Plant Sci.

[9] Yang BF, Li YN, Song Y, Wang XL, Guo QX, Zhou LX (2022). The R2R3-MYB transcription factor VcMYB4a inhibits lignin biosynthesis in blueberry (Vaccinium corymbosum). Tree Genet Genomes.

[10] Tanaka Y, Sasaki N, Ohmiya A (2008). Biosynthesis of plant pigments: anthocyanins, betalains and carotenoids. Plant J.

[11] Chen YT, Cheng CZ, Feng X, Lai RL, Gao MX, Chen WG (2021). Integrated analysis of lncRNA and protein-coding genes transcriptomes reveals the potential regulatory role of lncRNA in kiwifruit ripening and softening. Sci Rep.

[12] Zhang B, Su TB, Li PR, Xin XY, Cao YY, Wang WH (2021). Identification of long noncoding RNAs involved in resistance to downy mildew in Chinese cabbage. Hortic Res.

[13] Wu L, Liu SA, Qi HR, Cai H, Xu M (2020). Research progress on plant long non-coding RNA. Plants.

[14] Wu XX, Shi T, Iqbal S, Zhang Y, Liu L, Gao ZH (2019). Genome-wide discovery and characterization of flower development related long non-coding RNAs in Prunus mume. BMC Plant Biol.

[15] Gao C, Sun JL, Dong YM, Wang CQ, Xiao SH, Mo LF (2020). Comparative transcriptome analysis uncovers regulatory roles of long non-coding RNAs involved in resistance to powdery mildew in melon. BMC Genomics.

[16] Jain P, Hussian S, Nishad J, Dubey H, Bisht DS, Sharma TR (2021). Identification and functional prediction of long non-coding RNAs of rice (Oryza sativa L.) at reproductive stage under salinity stress. Mol Biol Rep.

[17] Zhao QH, Yang QY, Wang ZS, Sui Y, Wang Q, Liu J (2021). Analysis of long non-coding RNAs and protein-coding genes in harvested kiwifruit in response to the yeast antagonist Wickerhamomyces anomalus. Comput Struct Biotec.

[18] Cabili MN, Trapnell C, Goff L, Koziol M, Tazon-Vega B, Regev A (2011). Integrative annotation of human large intergenic noncoding RNAs reveals global properties and specific subclasses. Genes Dev.

[19] Liu NK, Xu YZ, Li Q, Cao YX, Yang DC, Liu SS (2022). A lncRNA fine-tunes salicylic acid biosynthesis to balance plant immunity and growth. Cell Host Microbe.

[20] Moison M, Pacheco JM, Lucero L, Fonouni-Farde C, Rodríguez-Melo J, Mansilla N (2021). The lncRNA APOLO interacts with the transcription factor WRKY42 to trigger root hair cell expansion in response to cold. Mol Plant.

[21] Yu JX, Qiu KN, Sun WJ, Yang T, Wu T, Song TT (2022). A long noncoding RNA functions in high-light-induced anthocyanin accumulation in apple by activating ethylene synthesis. Plant Physiol.

[22] Heo JB, Sung S (2011). Vernalization-mediated epigenetic silencing by a long intronic noncoding RNA. Science.

[23] Csorba T, Questa JI, Sun Q, Dean C (2014). Antisense COOLAIR mediates the coordinated switching of chromatin states at FLC during vernalization. Proc Natl Acad Sci U S A.

[24] Rai MI, Alam M, Lightfoot DA, Gurha P, Afzal AJ (2019). Classification and experimental identification of plant long non-coding RNAs. Genomics.

[25] Chen JJ, Zhong YQ, Qi X (2021). LncRNA TCONS_00021861 is functionally associated with drought tolerance in rice (Oryza sativa L.) via competing endogenous RNA regulation. BMC Plant Biol.

[26] Zhu CZ, Zhang ST, Fu HF, Zhou CZ, Chen L, Li XZ (2019). Transcriptome and phytochemical analyses provide new insights into long non-coding RNAs modulating characteristic secondary metabolites of oolong tea (Camellia sinensis) in solar-withering. Front Plant Sci.

[27] Song XM, Hu JJ, Wu T, Yang QH, Feng XH, Lin H, et al.. Comparative analysis of long noncoding RNAs in angiosperms and characterization of long noncoding RNAs in response to heat stress in Chinese cabbage. Hortic. Res. 2021;8. 10.1038/s41438-021-00484-4 PMC7917108 33642591

[28] Tian YY, Bai SL, Dang ZH, Hao JF, Zhang J, Hasi A (2019). Genome-wide identification and characterization of long non-coding RNAs involved in fruit ripening and the climacteric in Cucumis melo. BMC Plant Biol.

[29] Li HY, Lv QY, Liu AK, Wang JR, Sun XQ, Deng J (2022). Comparative metabolomics study of Tartary (Fagopyrum tataricum L. Gaertn) and common (Fagopyrum esculentum Moench) buckwheat seeds. Food Chem.

[30] Li ZX, Chen ZX, Tang JM, Jiang YS, Liao QH, Liu YQ, et al. Metabolomic analysis of bioactive compounds in mature rhizomes and daughter rhizomes in ginger (Zingiber officinale), 2019. 10.21203/rs.2.17463/v1

[31] Zhang W, Han ZX, Guo QL, Liu Y, Zheng YX, Wu FL (2014). Identification of maize long non-coding RNAs responsive to drought stress. PLoS ONE.

[32] Zhang GY, Chen DG, Zhang T, Duan AG, Zhang JG, He C (2018). Transcriptomic and functional analyses unveil the role of long non-coding RNAs in anthocyanin biosynthesis during sea buckthorn fruit ripening. DNA Res.

[33] Sahu S, Rao AR, Pandey J, Gaikwad K, Ghoshal S, Mohapatra T (2018). Genome-wide identification and characterization of lncRNAs and miRNAs in cluster bean (Cyamopsis tetragonoloba). Gene.

[34] Cheynier V, Comte G, Davies KM, Lattanzio V, Martens S (2013). Plant phenolics: recent advances on their biosynthesis, genetics, and ecophysiology. Plant physiol Bioch.

[35] Ma HY, Yang T, Li Y, Zhang J, Wu T, Song TT (2021). The long noncoding RNA MdLNC499 bridges MdWRKY1 and MdERF109 function to regulate early-stage light-induced anthocyanin accumulation in apple fruit. Plant Cell.

[36] Wang ZP, Liu YF, Li L, Li DW, Zhang Q, Guo YT (2017). Whole transcriptome sequencing of Pseudomonas syringae pv. actinidiae-infected kiwifruit plants reveals species-specific interaction between long non-coding RNA and coding genes. Sci Rep.

[37] Wang F, Ji GS, Xu ZB, Feng B, Zhou Q, Fan XL (2021). Metabolomics and transcriptomics provide insights into anthocyanin biosynthesis in the developing grains of purple wheat (Triticum aestivum L.). J Agr Food Chem.

[38] Kim D, Paggi JM, Park C, Bennett C, Salzberg SL (2019). Graph-based genome alignment and genotyping with HISAT2 and HISAT-genotype. Nature Biotechnol.

[39] Pertea M, Pertea GM, Antonescu CM, Chang TC, Mendell JT, Salzberg SL (2015). StringTie enables improved reconstruction of a transcriptome from RNA-seq reads. Nature Biotechnol.

[40] Kang YJ, Yang DC, Kong L, Hou M, Meng YQ, Wei L (2017). CPC2: a fast and accurate coding potential calculator based on sequence intrinsic features. Nucleic Acids Res.

[41] Trapnell C, Williams BA, Pertea G, Mortazavi A, Kwan G, Van Baren MJ (2010). Transcript assembly and quantification by RNA-Seq reveals unannotated transcripts and isoform switching during cell differentiation. Nature Biotechnol.

[42] Wang KL, Zhang Y, Zhang HM, Lin XC, Xia R, Song L (2021). MicroRNAs play important roles in regulating the rapid growth of the Phyllostachys edulis culm internode. New Phytol.

[43] Kanehisa M, Furumichi M, Sato Y, Kawashima M, Ishiguro-Watanabe M (2023). KEGG for taxonomy based analysis of pathways and genomes. Nucleic Acids Res.

