# Supplementary figures and images for: Integrated analyses of metabolomics and transcriptomics reveal the potential regulatory roles of long non-coding RNAs in gingerol biosynthesis
Source: BMC Genomics. 2023 Aug 26;24:490. doi: 10.1186/s12864-023-09553-5 (PMC10464350; doi:10.1186/s12864-023-09553-5)

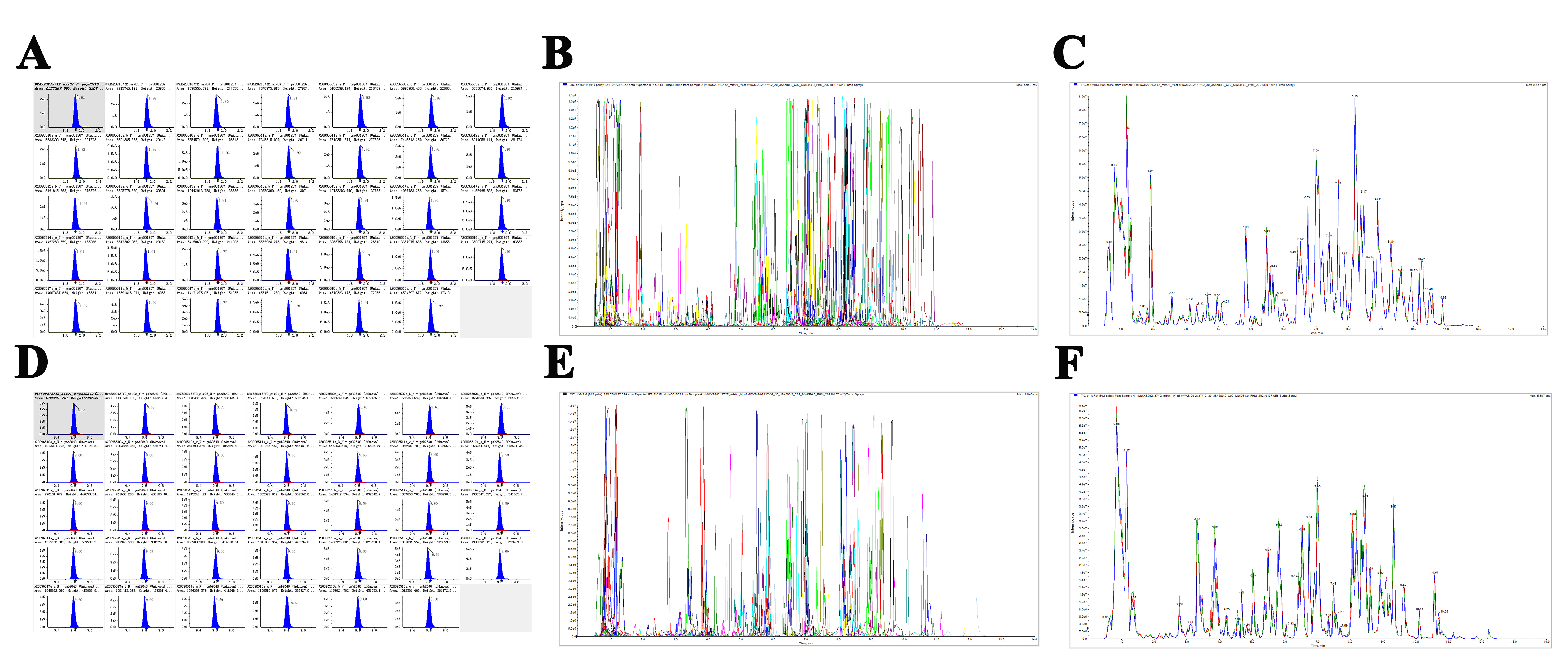

Supplement: Supplementary file 1 — Additional file 1: Fig. S1. Integration calibration analysis of randomly selected positive (A) and negative (D) ionization compounds in different samples; x-axis represents the retention time (min) of metabolite detection, and y-axis represents the ion current intensity (cps) of ion detection. Multimodal plot analysis of positive (B) and negative (E) ionization compounds by MRM metabolite detection. Total ion chromatogram of positive (C) and negative (F) ionization compounds in mixed samples by mass spectrometry analysis. [file 12864_2023_9553_MOESM1_ESM.jpg]

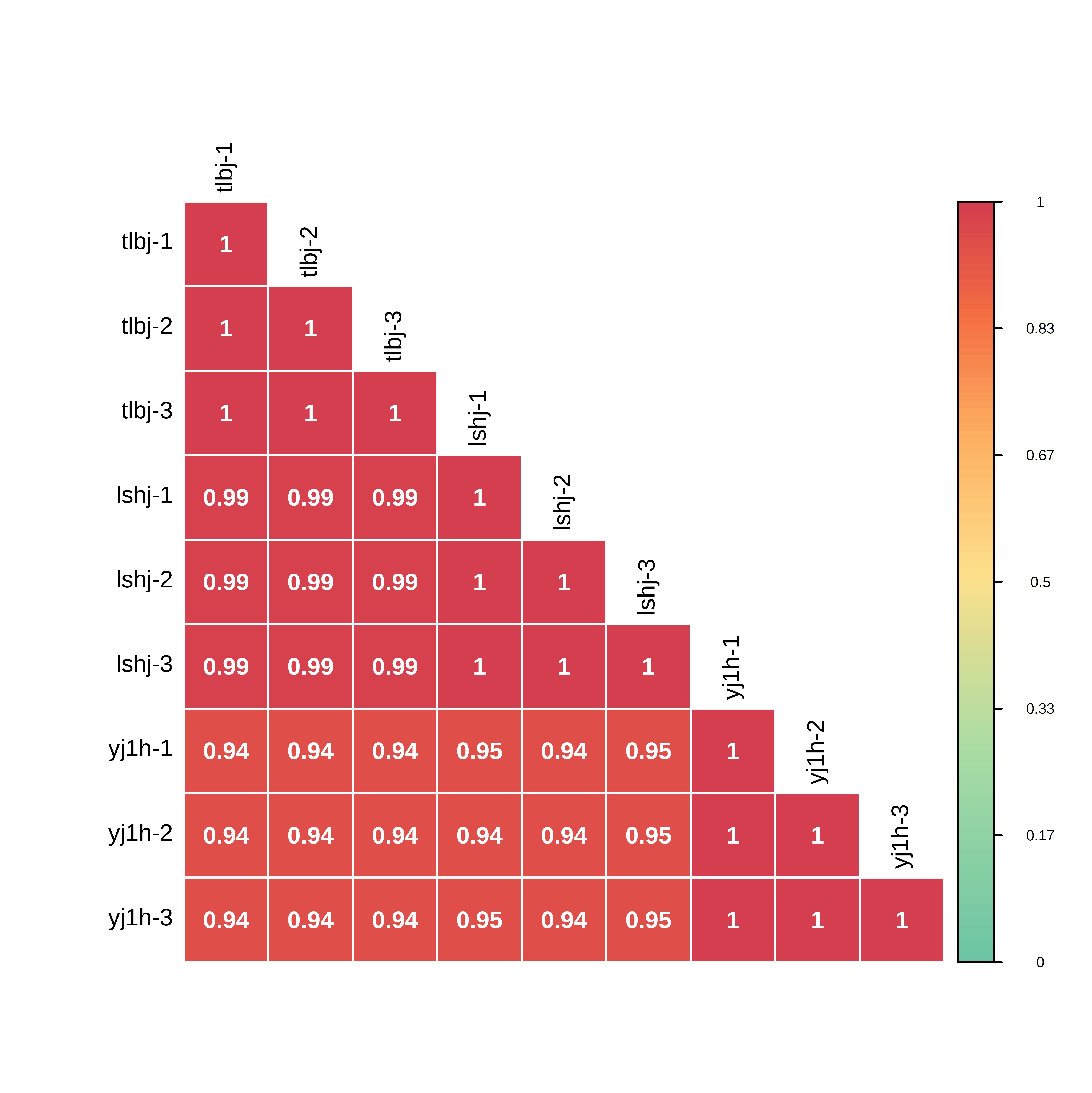

Supplement: Supplementary file 2 — Additional file 2: Fig. S2. Correlation analysis among the three replicates of the metabolome. [file 12864_2023_9553_MOESM2_ESM.jpg]

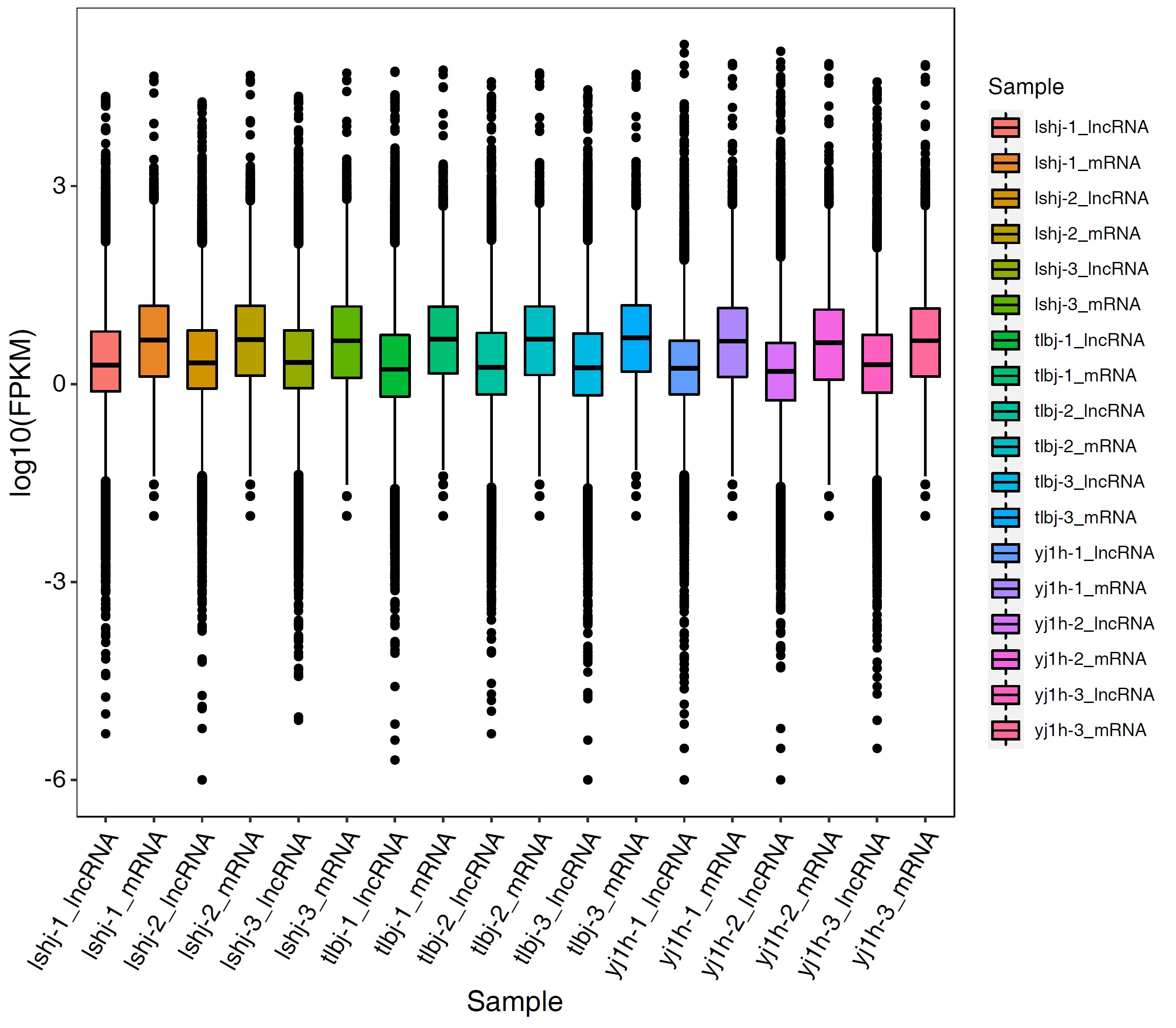

Supplement: Supplementary file 3 — Additional file 3: Fig. S3. The expression levels of lncRNA genes (A) and protein-coding mRNA genes (B) in the rhizomes of three ginger varieties. [file 12864_2023_9553_MOESM3_ESM.jpg]

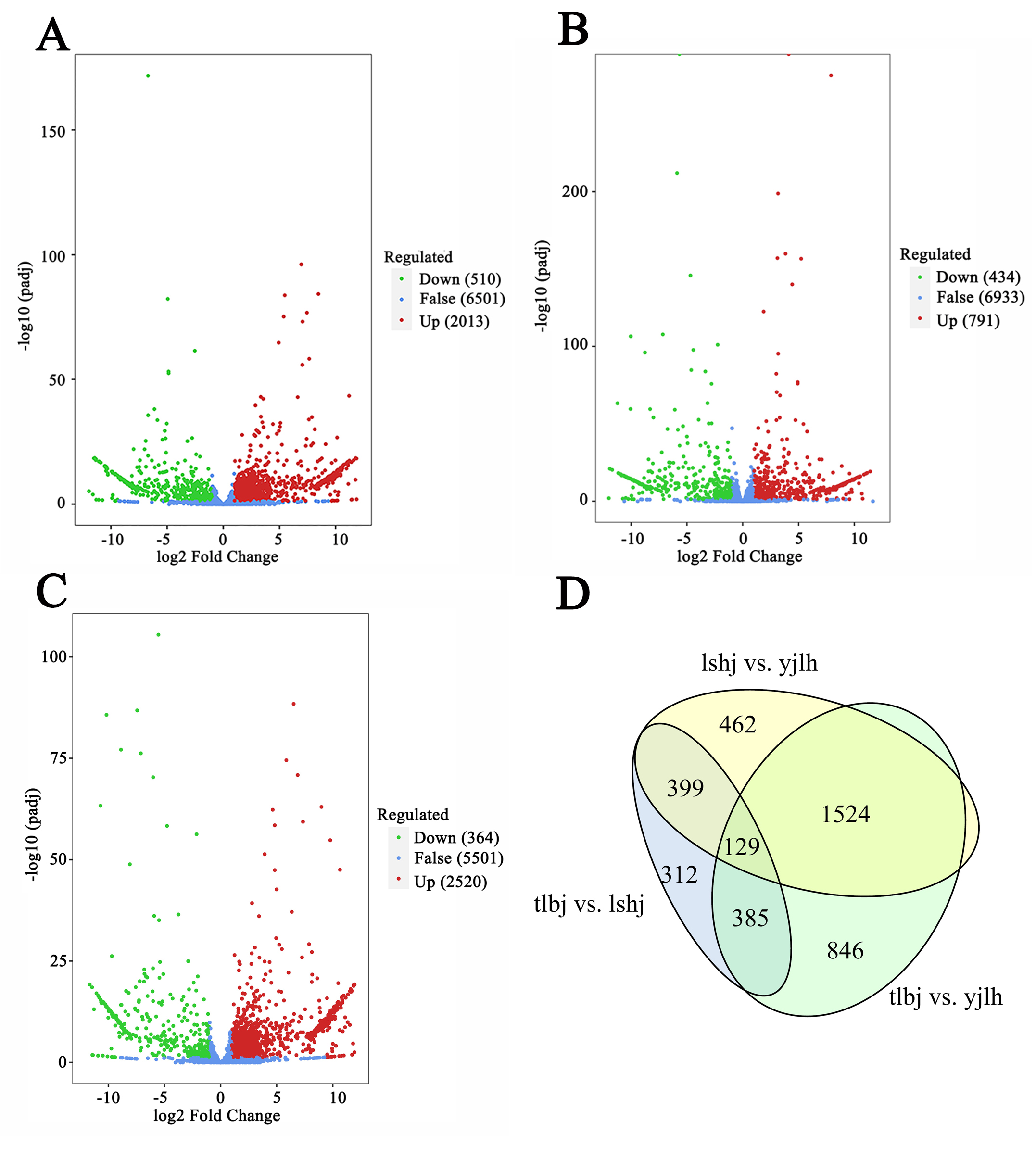

Supplement: Supplementary file 4 — Additional file 4: Fig. S4. The volcano figures for the DELs in the three comparisons of lshj vs. yj1h (A), tlbj vs. lshj (B), and tlbj vs. yj1h (C), respectively. (D) Venn diagram analysis of DELs shared among two or three comparisons. [file 12864_2023_9553_MOESM4_ESM.jpg]

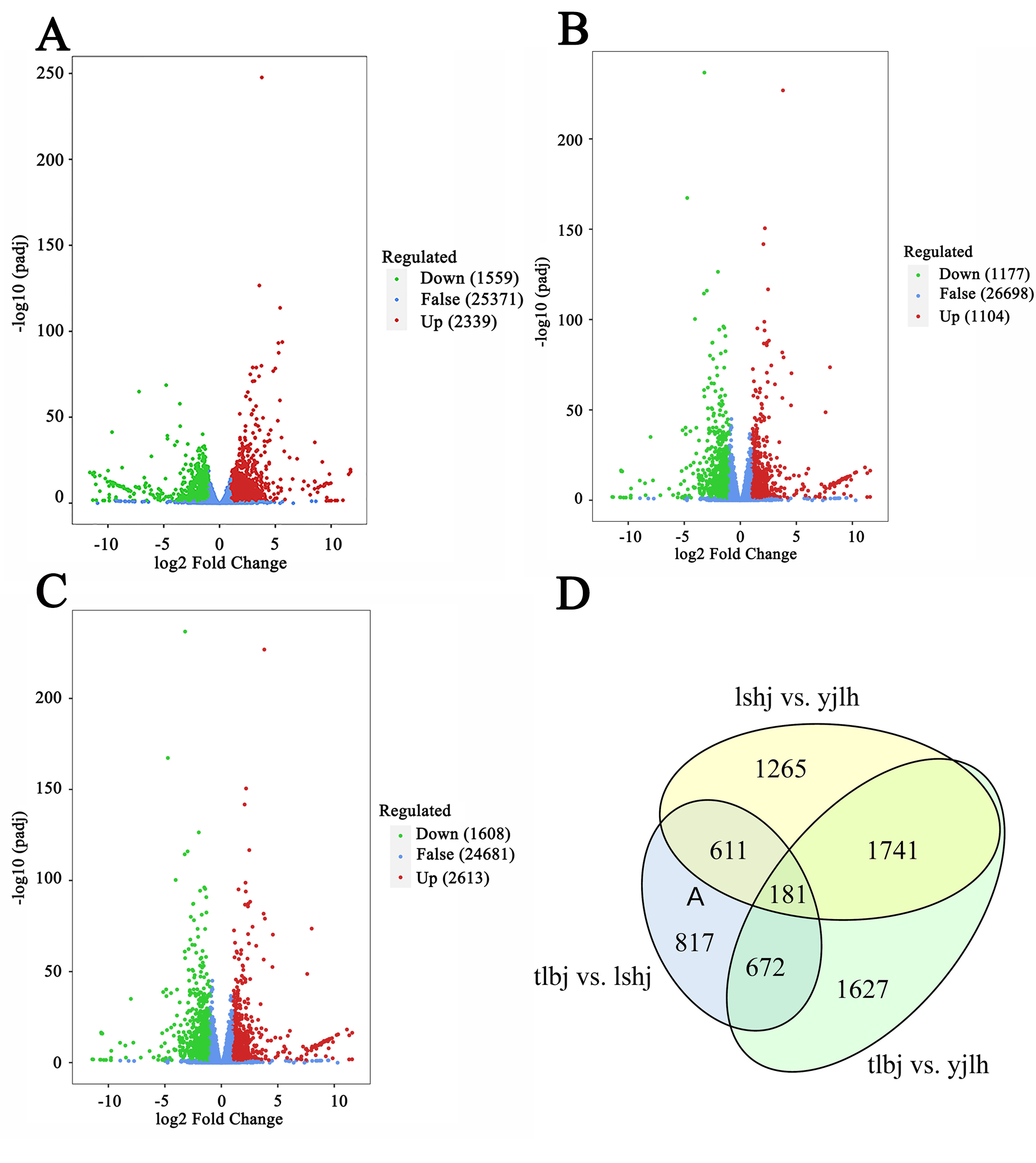

Supplement: Supplementary file 5 — Additional file 5: Fig. S5. The volcano figures for the DEPCGs in the three comparisons of lshj vs. yj1h (A), tlbj vs. lshj (B), and tlbj vs. yj1h (C), respectively. (D) Venn diagram analysis of DEGs shared among two or three comparisons. [file 12864_2023_9553_MOESM5_ESM.jpg]

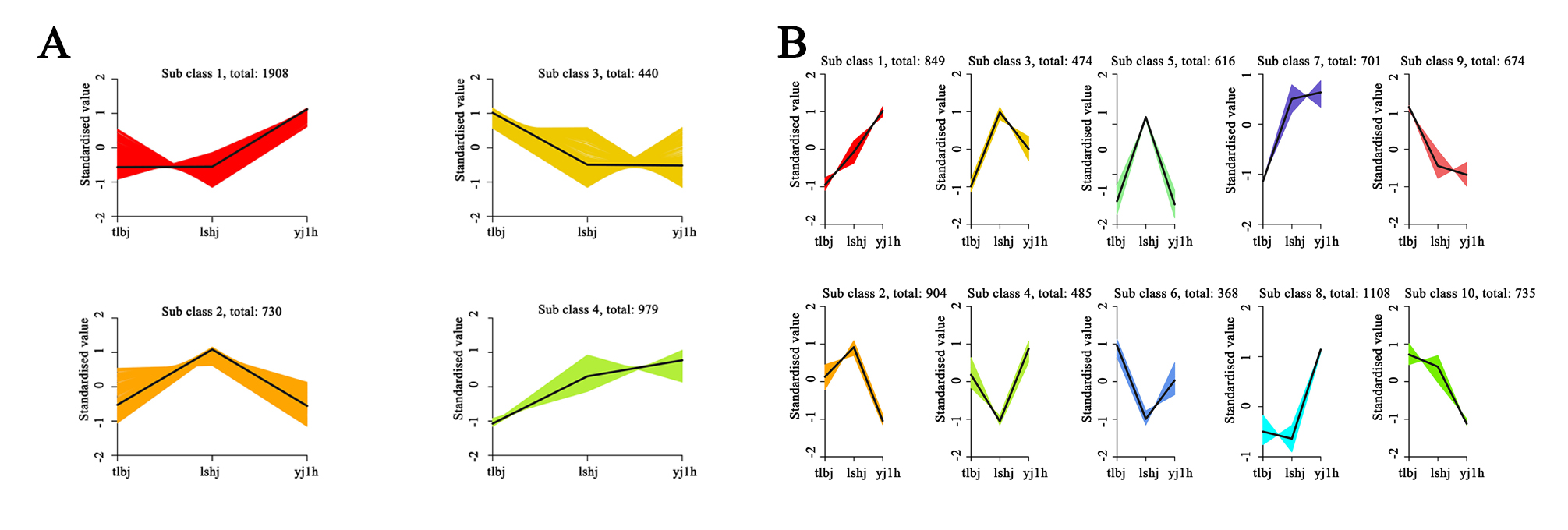

Supplement: Supplementary file 6 — Additional file 6: Fig. S6. K-means clustering groups of DELs (A) and DEPCGs (B) in the three ginger cultivars. The y-axis represents the standardized value per gene, and the x-axis represents the different cultivars. [file 12864_2023_9553_MOESM6_ESM.jpg]

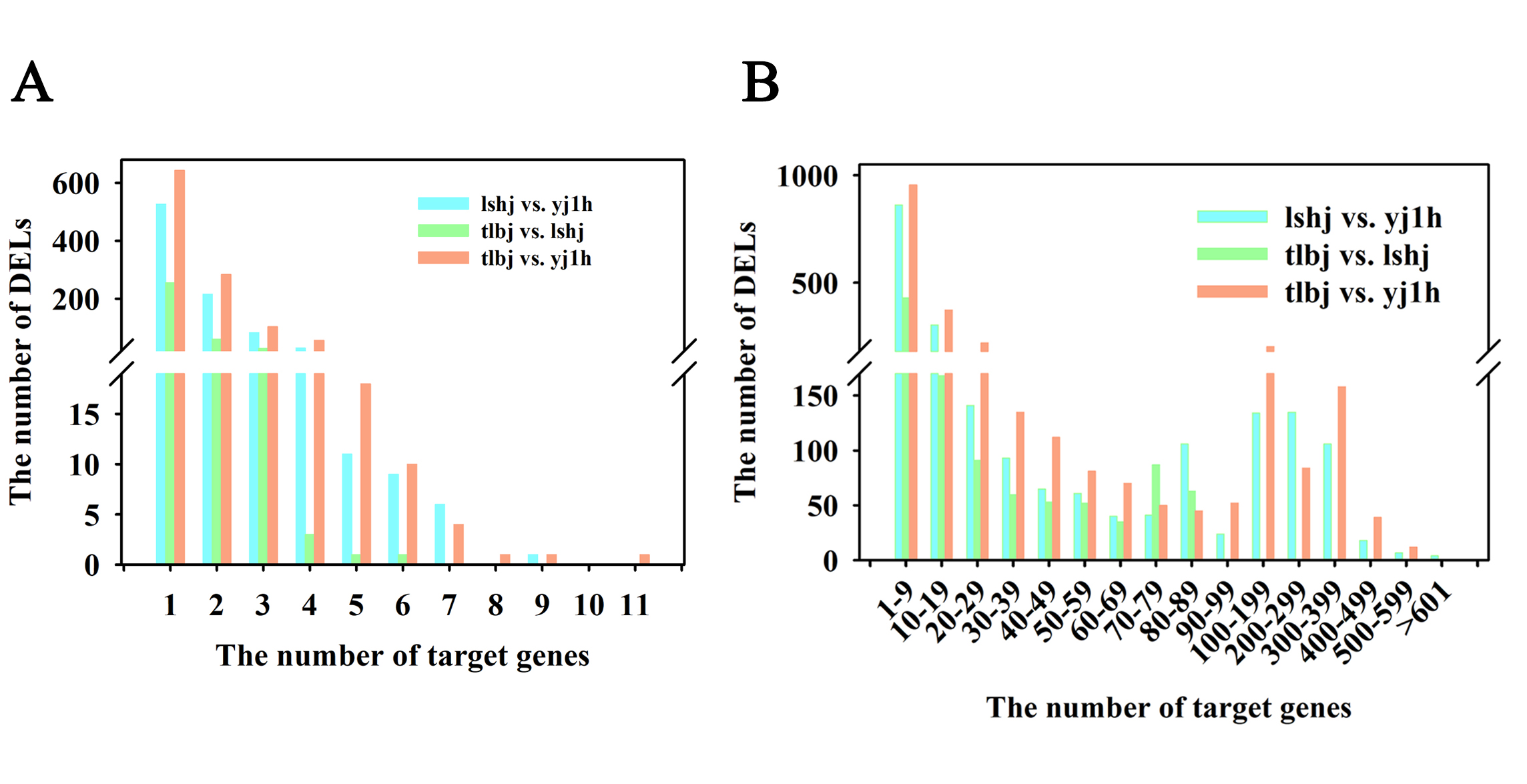

Supplement: Supplementary file 7 — Additional file 7: Fig. S7. (A) The DEPCGs targets of DELs in three comparisons under the cis-acting model. (B) The DEPCGs target DELs in three comparisons under the trans-acting model. [file 12864_2023_9553_MOESM7_ESM.jpg]

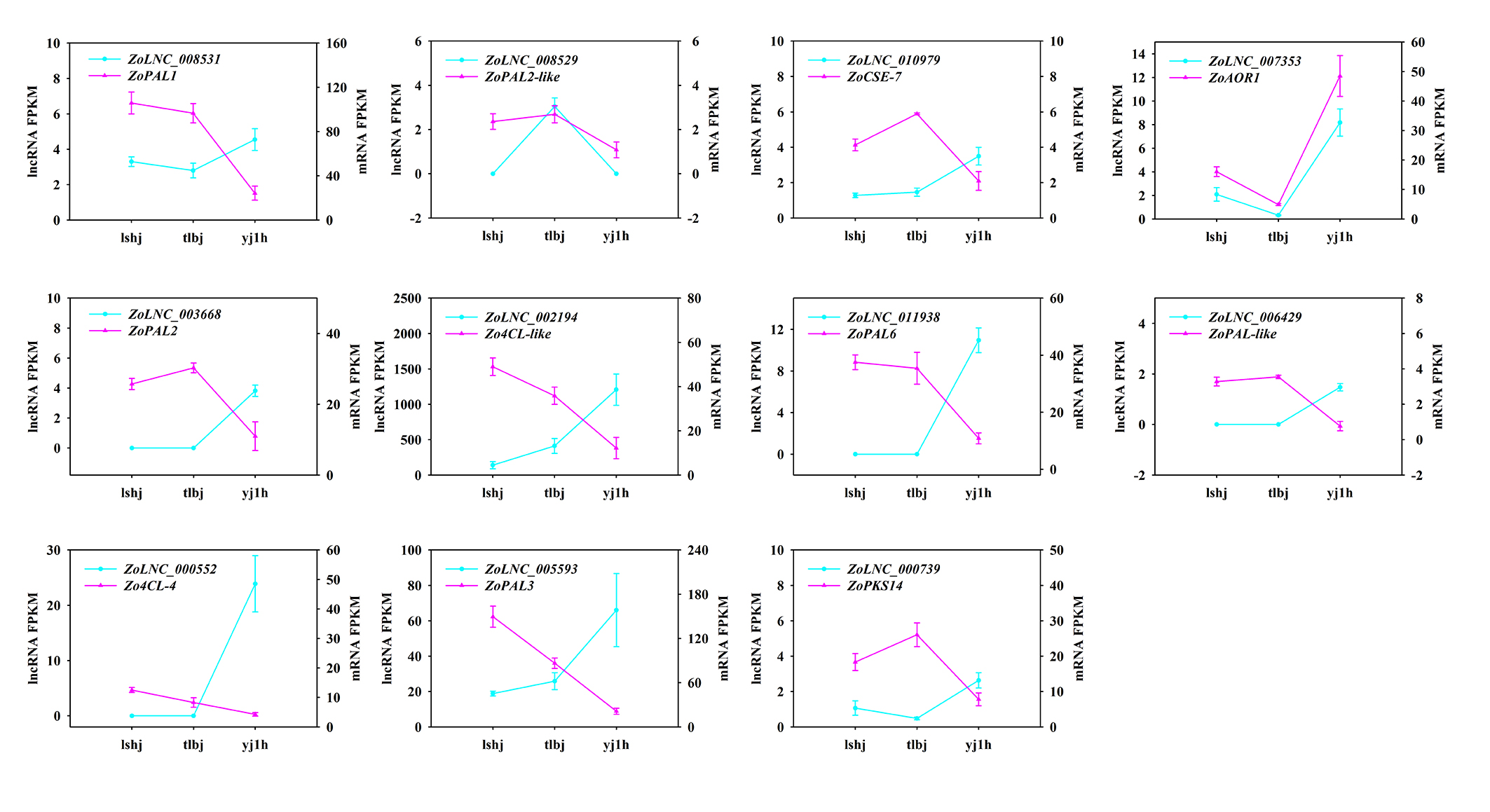

Supplement: Supplementary file 8 — Additional file 8: Fig. S8. The relative expression levels of 11 ginger biosynthesis genes and 11 corresponding lncRNAs in three ginger varieties by RNA-seq. [file 12864_2023_9553_MOESM8_ESM.jpg]

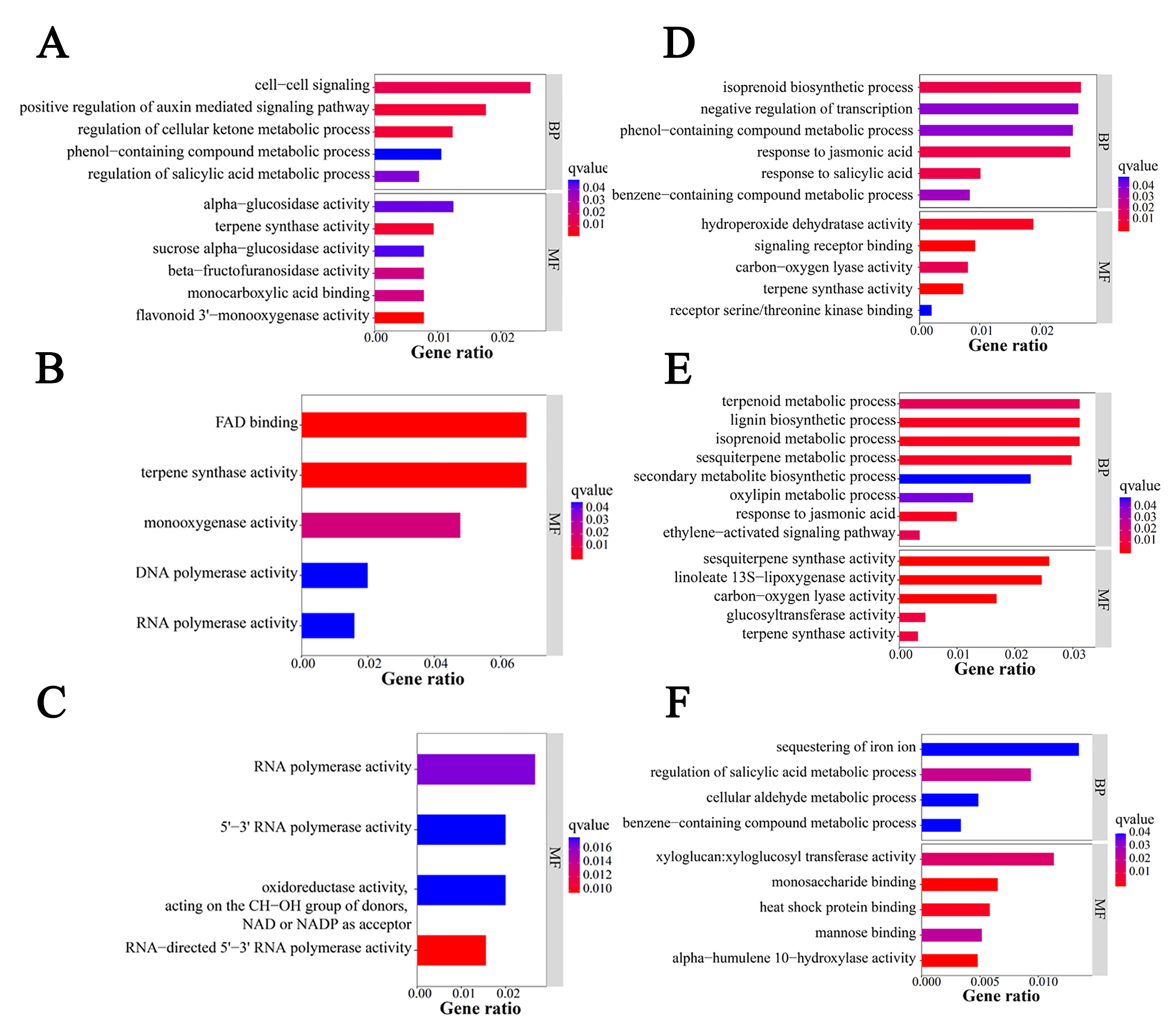

Supplement: Supplementary file 9 — Additional file 9: Fig. S9. Statistical analysis of GO enrichment of DELs based on the cis-acting model in the three comparisons of lshj vs. yj1h (A), tlbj vs. lshj (B), and tlbj vs. yj1h (C), or based on the trans-acting model in the three comparisons of lshj vs. yj1h (D), tlbj vs. lshj (E), and tlbj vs. yj1h (F). [file 12864_2023_9553_MOESM9_ESM.jpg]
